# Evolution-Informed Discovery of the Naphthalenone Biosynthetic Pathway in Fungi

**DOI:** 10.1128/mbio.00223-22

**Published:** 2022-05-26

**Authors:** Olga V. Mosunova, Jorge C. Navarro-Muñoz, Diksha Haksar, Jacq van Neer, Jelmer Hoeksma, Jeroen den Hertog, Jérôme Collemare

**Affiliations:** a Westerdijk Fungal Biodiversity Institute, Utrecht, The Netherlands; b Hubrecht Institute-KNAW, Utrecht, The Netherlands; c University Medical Center Utrecht, Utrecht, The Netherlands; d Institute Biology Leiden, Leiden University, Leiden, The Netherlands; Cornell University

**Keywords:** biosynthetic gene clusters, comparative genomics, phylogeny, heterologous expression, *Aspergillus parvulus*, *Aspergillus oryzae*, lichen, polyketide, nonreducing polyketide synthase, acetyl tetrahydroxynaphthalene

## Abstract

Fungi produce a wide diversity of secondary metabolites with interesting biological activities for the health, industrial, and agricultural sectors. While fungal genomes have revealed an unexpectedly high number of biosynthetic pathways that far exceeds the number of known molecules, accessing and characterizing this hidden diversity remain highly challenging. Here, we applied a combined phylogenetic dereplication and comparative genomics strategy to explore eight lichenizing fungi. The determination of the evolutionary relationships of aromatic polyketide pathways resulted in the identification of an uncharacterized biosynthetic pathway that is conserved in distant fungal lineages. The heterologous expression of the homologue from Aspergillus parvulus linked this pathway to naphthalenone compounds, which were detected in cultures when the pathway was expressed. Our unbiased and rational strategy generated evolutionary knowledge that ultimately linked biosynthetic genes to naphthalenone polyketides. Applied to many more genomes, this approach can unlock the full exploitation of the fungal kingdom for molecule discovery.

## INTRODUCTION

Fungi are an illustrious source of small bioactive compounds, called natural products or secondary metabolites (SMs), which are not strictly required for growth and reproduction. Instead, they serve as chemical mediators of interactions with the physical environment and with other organisms. At present, fungal SMs are experiencing a renewed interest in drug discovery after the high-throughput screening of libraries of synthetic compounds showed its limitation in yielding new bioactive molecules (1). Fungi have provided us with key antimicrobial compounds, like the first broad-spectrum antibiotic, penicillin, and the first antifungal compound of the caspofungin class, demonstrating their life-changing potential (2, 3). The genomic era has revealed that the fungal kingdom has been underexploited because fungal genomes encode an outstanding number of biosynthetic pathways that is far higher than the number of known fungal molecules (4).

The ever-increasing number of fungal genomes provides an exciting opportunity to identify novel bioactive molecules, but at the same time, such a vast amount of data represents a significant challenge to successful exploitation. So far, analyses of fungal genomes have mostly been restricted to searching the genes involved in the production of already known molecules (5–8) or to surveying the global biosynthetic potential of a given fungus or fungal lineage (9–11). While providing interesting frameworks for functional analyses, such approaches do not allow the rational exploitation of fungal genomes for molecule discovery.

Phylogenetic studies have revealed that closely related SM biosynthetic enzymes tend to share similar catalytic activities and produce similar chemical backbones (12, 13). Prioritizing functional studies using so-called phylogenetic dereplication to discover novel backbones have been rudimentarily employed only. Using this approach, Harvey and coworkers selected 41 biosynthetic pathways from diverse fungal species for functional studies, leading to the detection of 22 compounds, including a few novel ones (14). This previous report showed the potential of this approach to prioritize functional analyses for molecule discovery, but it did not make full use of the evolutionary information underlying phylogenetic relationships, and it did not make use of comparative genomics. Comparing evolutionarily related biosynthetic pathways is necessary to resolve complex metabolic patterns in distinct fungi. Thus, evolution-informed analysis of fungal biosynthetic pathways is still an unexplored ground for molecule discovery and the full exploitation of fungal genomes (15).

Fungal SMs are highly structurally diverse and exhibit various biological activities, yet SM biosynthesis relies on a few classes of core enzymes, including polyketide synthases (PKSs), nonribosomal peptide synthetases (NRPSs), and terpene cyclases (TCs) (16). In addition to the core enzyme, the biosynthesis of a given SM typically involves other so-called tailoring enzymes, which are encoded by genes that are usually organized into biosynthetic gene clusters (BGCs), meaning that they colocalize in the genome and are coregulated (16). Within the fungal kingdom, the Ascomycota show the greatest potential, with 12 to 68 BGCs on average per fungal genome (17). Within the Ascomycota, the Lecanoromycetes class is particularly interesting because it comprises species with the highest number of PKSs per genome. Most known polyketides from the Lecanoromycetes are structurally related and comprise an orsellinic acid-like backbone, as in depsides (atranorin, lecanoric acid, and sekikaic acid), depsidones (lobaric acid), and depsones (picrolichenic acid) (18). Other known Lecanoromycetes polyketides exhibit chemical structures like anthra- and naphthoquinones (parietin, rhodocladonic acid, and cristazarin), dibenzofurans (usnic acid and pannaric acid), or chromones (lepraric acid) (19). Despite many known compounds, to date, only atranorin and lecanoric acid have been functionally linked to their respective BGCs (20, 21). A few compounds have been putatively assigned to BGCs based on genomic and transcriptomic information (6, 21–23), but they remain to be functionally validated, and thus, the vast majority of lichen compounds remain unassigned to BGCs. In addition, genetic and chemoinformatic analyses of BGCs in Ascomycota fungi suggest that the Lecanoromycetes comprise a reservoir of fairly dissimilar biosynthetic pathways compared to other classes of the Ascomycota, and therefore, their genetic and chemical potential has remained far from characterized (9).

In this study, we present how an evolution-informed strategy that combines phylogenetic dereplication and comparative genomics can be used to prioritize BGC functional characterization, link BGCs to molecules, and, ultimately, fully exploit fungal chemical diversity. The phylogenetic dereplication of nonreducing polyketide synthases (nrPKSs) encoded in eight Lecanoromycetes genomes revealed a novel biosynthetic pathway in Lobaria pulmonaria and Umbilicaria pustulata lichen mycobionts. A comparative genomics approach identified a homologous pathway in Aspergillus parvulus, and its nrPKS was functionally characterized using heterologous expression in Aspergillus oryzae. Thanks to the determination of the evolutionary relationships between polyketide BGCs, we were able to predict chemical diversity in distinct fungal lineages, and we suggest that L. pulmonaria and U. pustulata may produce SMs unreported for these species, which might play a role during their interaction with their respective photobionts.

## RESULTS

### Mining and phylogenetic dereplication of biosynthetic pathways encoded in Lecanoromycetes genomes identify an uncharacterized aromatic polyketide pathway.

**FIG 1:**
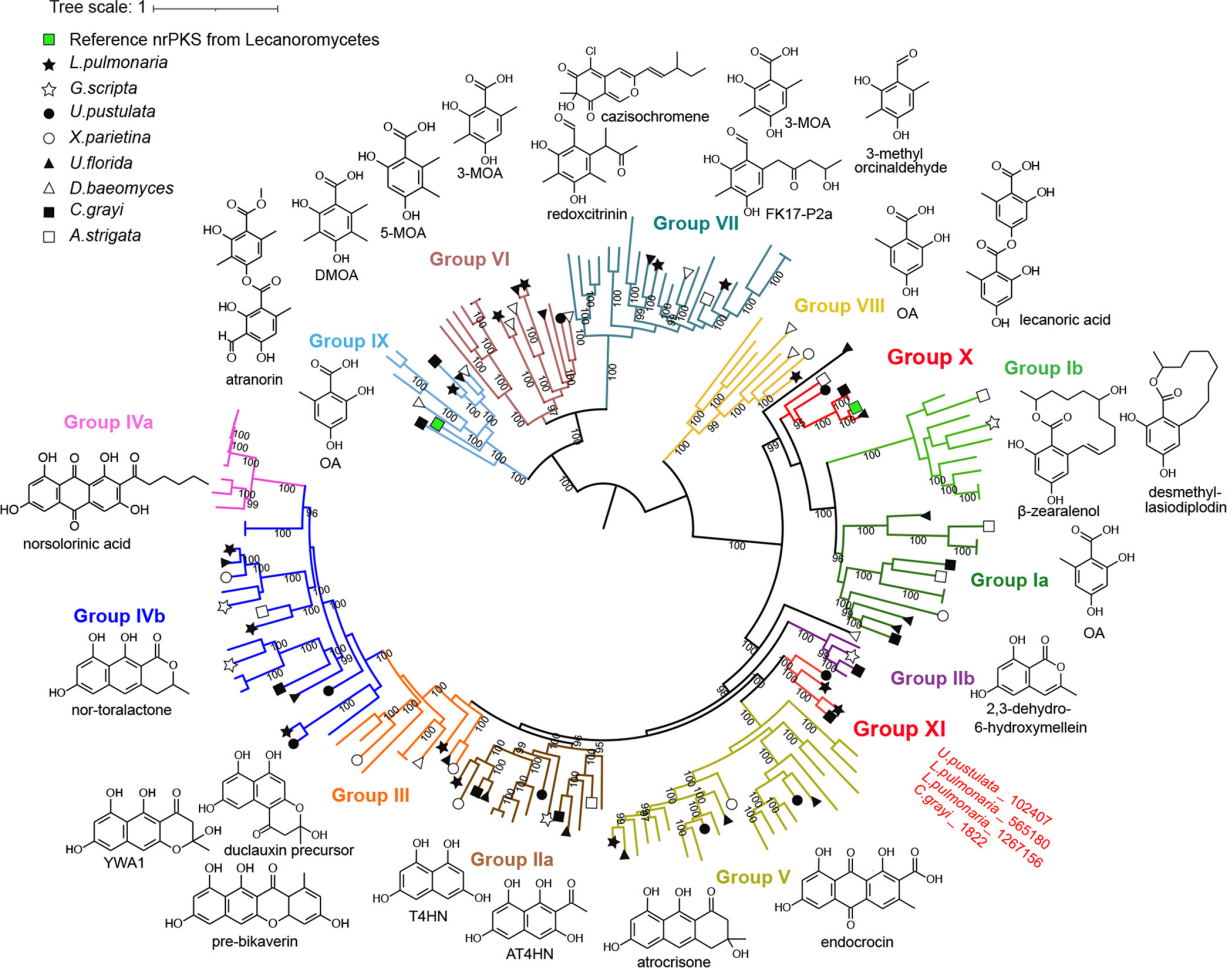
Phylogenetic dereplication of nonreducing polyketide synthases (nrPKSs) from eight Lecanoromycetes. A maximum likelihood phylogenetic tree was built with reference characterized nrPKSs from the MIBiG database (24) and from the literature (see Data Set S4 in the supplemental material). Ultrafast bootstrap values of >95 are shown. The tree is midpoint rooted. The nine phylogenetic groups as reported in previous analyses are indicated, with backbone molecules produced by the characterized nrPKSs shown next to each clade. Two new phylogenetic groups, X and XI, are highlighted in red. DMOA, 3,5-demethylorsellinic acid; MOA, methylorsellinic acid; OA, orsellinic acid; T4HN, 1,3,6,8-tetrahydroxynaphthalene; AT4HN, 2-acetyl-1,3,6,8-tetrahydroxynaphthalene; YWA1, 2,5,6,8-tetrahydroxy-2-methyl-2,3-dihydro-4H-naphtho[2,3-b]pyran-4-one.

Although Lecanoromycetes fungi have been extensively screened for bioactive molecules, most of these compounds belong to very few polyketide chemical classes, and the few published genome analyses indicated an unexpectedly high potential to produce diverse polyketides (9). While this potential has been explored to link BGCs to known molecules, it has not been used to identify novel biosynthetic pathways. For this purpose, a maximum likelihood phylogenetic tree of 79 predicted nrPKSs retrieved from eight Lecanoromycetes genomes (Cladonia grayi, Xanthoria parietina, Usnea florida, Lobaria pulmonaria, Acarospora strigata, Dibaeis baeomyces, Graphis scripta, and Umbilicaria pustulata) was built together with 87 characterized nrPKSs from the Minimum Information about a Biosynthetic Gene Cluster (MIBiG) database (24) and from the literature (Fig. 1; see also Data Sets S1 to S3 in the supplemental material).

10.1128/mbio.00223-22.6 DATA SET S1 Protein sequences of nonreducing polyketide synthases, hydroxynaphthalene reductases, and scytalone dehydratases. Download Data Set S1, TXT file, 0.4 MB. Copyright © 2022 Mosunova et al. 2022 Mosunova et al. https://creativecommons.org/licenses/by/4.0/ This content is distributed under the terms of the Creative Commons Attribution 4.0 International license.

10.1128/mbio.00223-22.7 DATA SET S2 Protein alignments of nonreducing polyketide synthases, hydroxynaphthalene reductases, and scytalone dehydratases. Download Data Set S2, TXT file, 0.4 MB. Copyright © 2022 Mosunova et al. 2022 Mosunova et al. https://creativecommons.org/licenses/by/4.0/ This content is distributed under the terms of the Creative Commons Attribution 4.0 International license.

10.1128/mbio.00223-22.8 DATA SET S3 Phylogenetic trees of nonreducing polyketide synthases, hydroxynaphthalene reductases, and scytalone dehydratases. Download Data Set S3, TXT file, 0.03 MB. Copyright © 2022 Mosunova et al. 2022 Mosunova et al. https://creativecommons.org/licenses/by/4.0/ This content is distributed under the terms of the Creative Commons Attribution 4.0 International license.

10.1128/mbio.00223-22.9 DATA SET S4 Curated gene models. Download Data Set S4, TXT file, 0.1 MB. Copyright © 2022 Mosunova et al. 2022 Mosunova et al. https://creativecommons.org/licenses/by/4.0/ This content is distributed under the terms of the Creative Commons Attribution 4.0 International license.

The tree is divided into 13 strongly supported clades, 8 of which correspond to previously defined nrPKS groups based on phylogeny and cyclization patterns (12, 21, 25). However, our analyses indicate that groups I, II, and IV should each be split into two subgroups that are consistent with the precursors released from the nrPKSs (Fig. 1). All nrPKS groups (IIa, IIb, III, IVa, IVb, and V) that produce polyketides with two or more aromatic rings share an origin, while other nrPKS groups that produce polyketides with a single aromatic ring seem to form three different clades (groups Ia and Ib; group VIII; and groups VI, VII, and IX). The most common Lecanoromycetes compounds, depsides, depsidones, depsones, and dibenzofurans, are thus likely produced by such single-ring-polyketide-producing nrPKSs. Consistently, the nrPKS involved in the production of atranorin belongs to group IX (21). Anthraquinones and xanthones like parietin and lichexanthone are likely produced by nrPKSs from group V because this clade comprises characterized nrPKSs involved in the biosynthesis of emodin-derived anthraquinones (Fig. 1).

In addition to these nine previously reported groups, two additional phylogenetic clades are strongly supported and are referred to as new phylogenetic groups X and XI (Fig. 1). Group X appears basal to groups Ia and Ib and thus likely comprises enzymes that produce polyketides with a single aromatic ring. The nrPKS involved in the production of the depside lecanoric acid in Pseudevernia furfuracea belongs to this clade (20), confirming that nrPKSs in this group produce orsellinic acid derivatives. In contrast, group XI does not comprise any characterized nrPKS. The basal position to group V suggests that the polyketides released by nrPKSs from group XI contain several aromatic rings. This group contains only four nrPKSs from *L. pulmonaria*, C. grayi, and *U. pustulata*, but manual curation of gene models revealed that *Clagr3_1822* is actually a pseudogene because it contains a disruptive mutation (Data Set S4). A single nrPKS from D. baeomyces is not related to any group and forms an outgroup to groups II to V and XI. Based on this phylogenetic dereplication, the vast majority of nrPKSs in the Lecanoromycetes fall into groups for which the chemical backbone can be predicted, which will be useful to assign known molecules to BGCs. We then embarked on characterizing the new group XI of fungal nrPKSs as it may be involved in the production of new polyketides in lichenizing and other fungi.

### Group XI nrPKSs belong to a novel conserved biosynthetic gene cluster.

**FIG 2:**
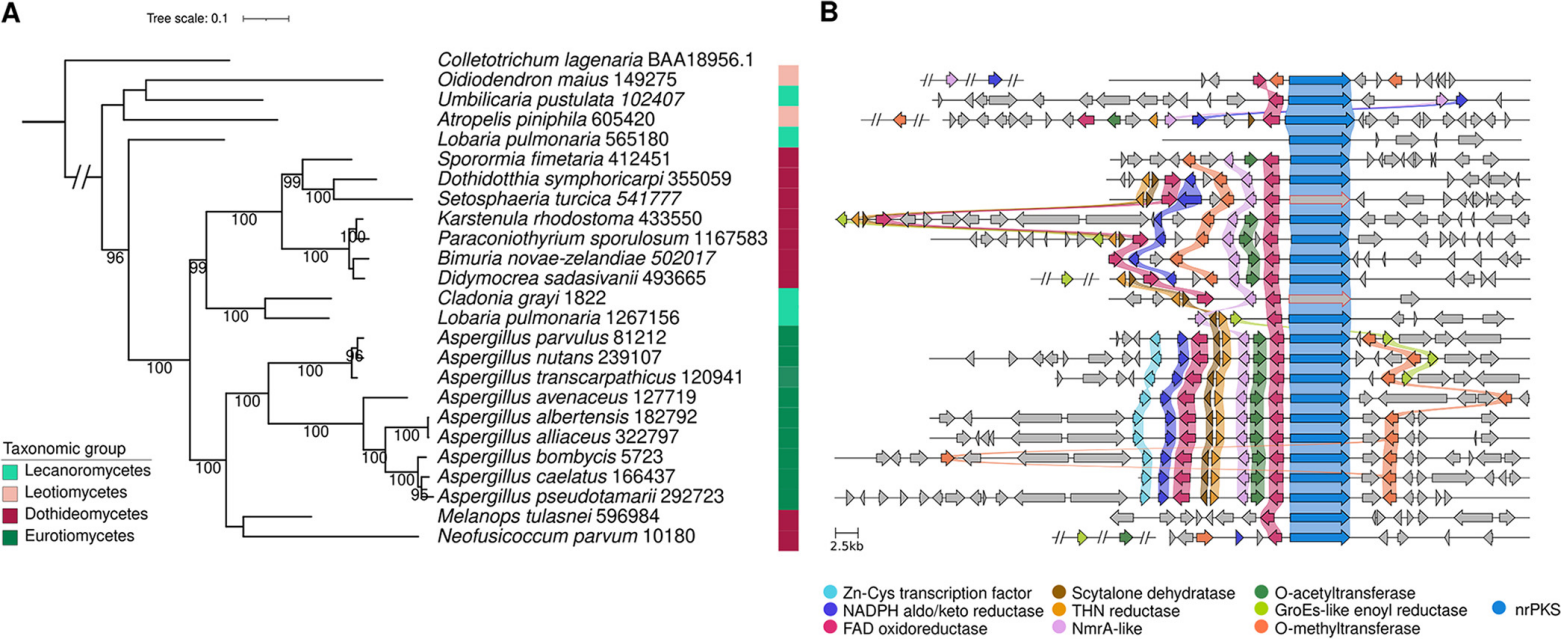
A novel biosynthetic gene cluster (BGC) from *Lobaria pulmonaria* and *Umbilicaria pustulata* is conserved in distant fungal species. (A) A maximum likelihood phylogenetic tree was built with homologues of group XI nonreducing polyketide synthases (nrPKSs), and the melanin nrPKS from Colletotrichum lagenarium was used as an outgroup. Ultrafast bootstrap values of >95 are shown. (B) Loci of nrPKS genes as predicted by fungiSMASH (42) were compared using Clinker (51). Colored arrows and connections show conserved genes that are predicted to be part of the BGC. Orange nrPKS arrows correspond to genes with disruptive mutations (see Table S1 in the supplemental material). Homologous genes that were found at another genomic location are depicted with double slashes. THN, tetrahydroxynaphthalene.

Because the phylogenetic dereplication was performed with functionally characterized nrPKSs only, it was not known whether group XI nrPKSs are restricted to the Lecanoromycetes or are present in other distant fungal species. To answer this question, we sought close homologues of the four group XI nrPKSs in available Ascomycota predicted proteomes. In total, we identified 20 other closely related nrPKSs, which expanded group XI to distant fungal lineages, including Leotiomycetes, Dothideomycetes, and Eurotiomycetes (Fig. 2A and Data Sets S1 to S3). The phylogeny of these homologues indicates that Lobpul1_1267156, Lobpul1_565180, and Umbpus1_102407 are actually paralogues (Fig. 2A). The Lobpul1_1267156 clade is further divided into two well-supported branches, both of which comprise Dothideomycetes sequences, also suggesting paralogy or horizontal transfer from Eurotiomycetes to Dothideomycetes (Fig. 2A).

10.1128/mbio.00223-22.4 TABLE S1 BLASTp search for proteins from the Aspergillus parvulus group XI biosynthetic pathway. Download Table S1, XLSX file, 0.03 MB. Copyright © 2022 Mosunova et al. 2022 Mosunova et al. https://creativecommons.org/licenses/by/4.0/ This content is distributed under the terms of the Creative Commons Attribution 4.0 International license.

We then compared the genomic loci of all *Lobpul1_1267156* homologues in order to predict the borders of a putative conserved BGC. Genes that encode putative tailoring enzymes were identified, based on their functional conserved domains, at all loci but one (Fig. 2B and Table S1). Although the *Lobpul1_565180* paralogue is predicted to be functional, this locus does not comprise any putative tailoring gene. A flavin adenine dinucleotide (FAD)-binding oxidoreductase tailoring gene is located upstream of the nrPKS gene in all species, and both genes appear to share a bidirectional promoter, except in Oidiodendron maius, in which an *O*-methyltransferase gene is inserted in between (Fig. 2B). A set of six other tailoring genes (encoding an *O*-acyltransferase, an *O*-methyltransferase, a tetrahydroxynaphthalene [T4HN] reductase, a scytalone dehydratase, an aldo-keto reductase, and a second FAD-binding oxidoreductase) and one putative regulatory gene encoding an NmrA-like protein are conserved in most of the fungal species and form a predicted BGC (Fig. 2B and Table S1). In addition, a gene encoding a GroES-like alcohol dehydrogenase enzyme is present at the locus in eight distant species, and a close homologue was found at a different locus in Didymocrea sadasivanii and Neofusicoccum parvum (Fig. 2B and Table S1). Similarly, close homologues of the aldo-keto reductase and NmrA-like protein-encoding genes were found at another locus in O. maius. Close homologues of the *O*-methyltransferase and *O*-acyltransferase genes are also found at a different locus in Atropellis piniphila and N. parvum, respectively. In Aspergillus species exclusively, a gene encoding a putative transcription factor is also found at the locus. Disruptive mutations were detected in the nrPKS gene not only in *C. grayi* but also in Setosphaeria turcica, suggesting a nonfunctional pathway in both species (Fig. 2B, Table S1, and Data Set S4). Similarly, disruptive mutations were found in a few tailoring genes, including the T4HN reductase and scytalone dehydratase genes in *L. pulmonaria*, and the insertion of a long sequence in an intron of the keto reductase gene in S. turcica likely makes it nonfunctional (Table S1 and Data Set S4). Although the phylogeny suggests a complex evolutionary history with several paralogues, the comparative genomics analysis indicates that nrPKS group XI belongs to a conserved BGC with little diversification between fungal species.

### Ancestral duplication of T4HN reductase and scytalone dehydratase genes.

**FIG 3:**
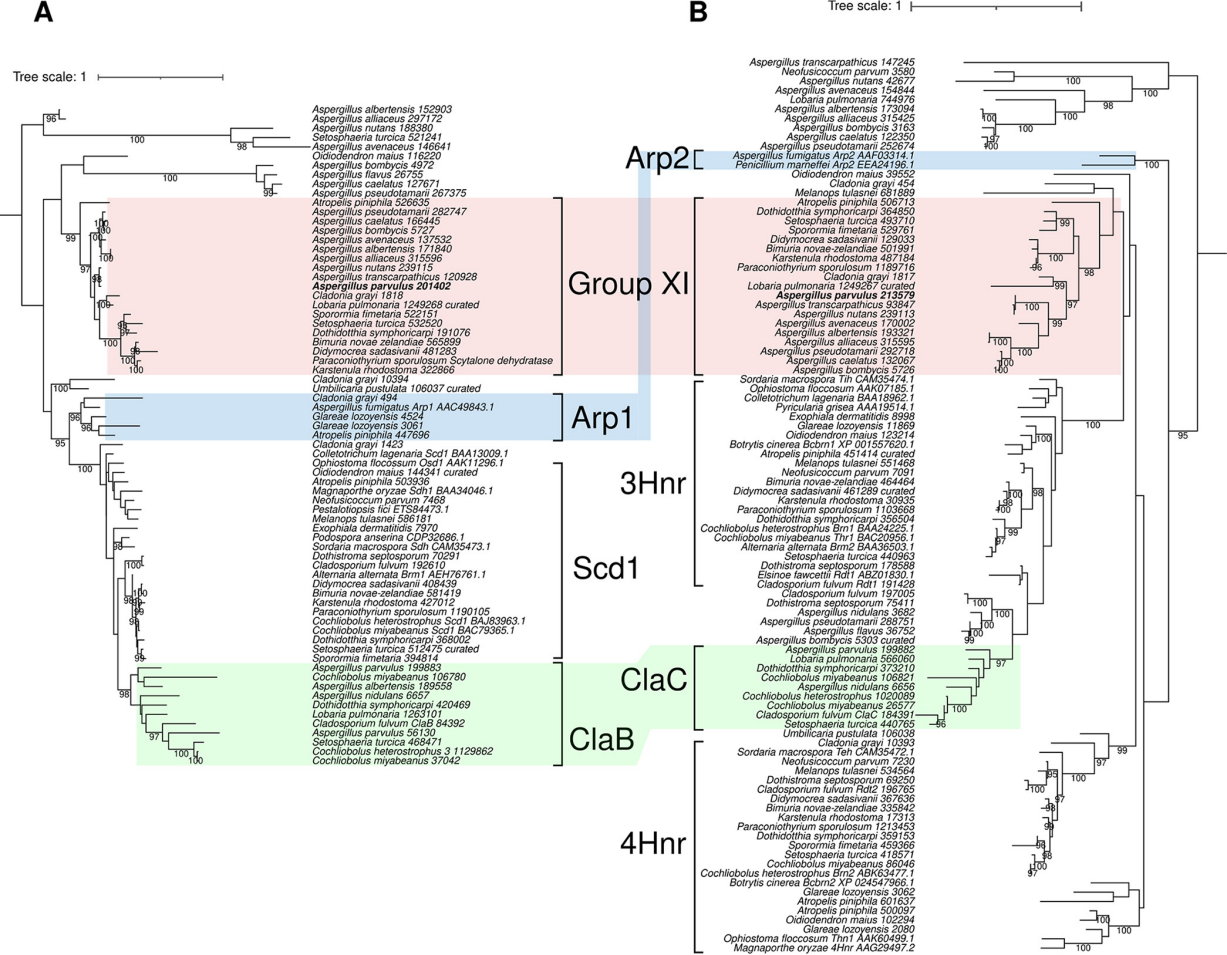
Coevolution between hydroxynaphthalene reductases (Hnr) and scytalone dehydratases. Maximum likelihood phylogenetic trees of homologues of scytalone dehydratases (A) and tetra- and tri-Hnr proteins (B) are shown (52). Paralogues that share a bidirectional promoter in the group XI, DHN melanin, and cladofulvin biosynthetic gene clusters (BGCs) are highlighted. Ultrafast bootstrap values of >95 are shown.

Two tailoring genes from the predicted group XI BGC encode a T4HN reductase and a scytalone dehydratase. These two enzymes are well characterized and act together to dehydroxylate intermediates in the conserved dihydroxynaphthalene (DHN) melanin (26) and anthraquinone cladofulvin (27) biosynthetic pathways. Especially, both genes in group XI appear to share a bidirectional promoter (Fig. 2B), a gene organization also found in the Aspergillus fumigatus DHN (*ARP1* and *ARP2*) (28, 29) and Cladosporium fulvum cladofulvin (27) BGCs, suggesting a common origin. A previous report indicated that *ARP1* and *ARP2* are distant paralogues of *claB* and *claC* that have been recruited in different biosynthetic pathways (30). Phylogenetic analyses of these enzymes, including the group XI sequences, are consistent with these previous findings (Fig. 3 and Data Sets S1 to S3) and reveal that group XI T4HN reductase and scytalone dehydratase are distinct paralogues with a similar evolutionary history. While *claB* and *claC* are most closely related to *SCD1* and *3HNR*, respectively, from the DHN melanin pathway found in Dothideomycetes and Sordariomycetes, the group XI gene pair could originate from an ancestral duplication of the *ARP1*-*ARP2* gene pair (Fig. 3).

### Aspparv1_81212 is a hexaketide synthase that produces AT4HN.

**FIG 4:**
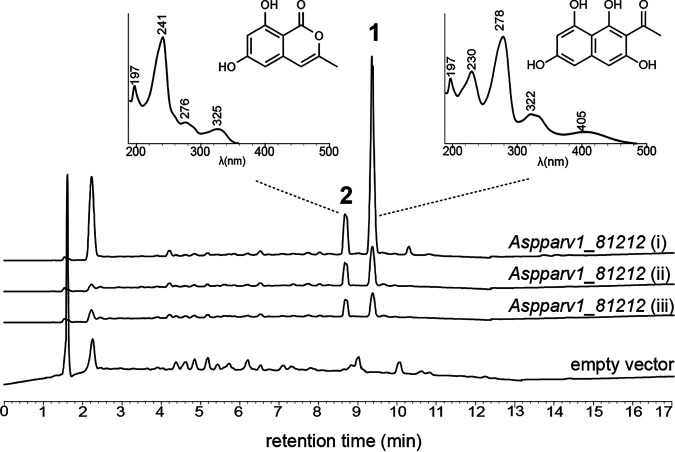
Heterologous expression of the group XI nonreducing polyketide synthase from Aspergillus parvulus. Organic extracts of 4-day-old Aspergillus oryzae NSAR1 transformants carrying an empty vector or expressing *Aspparv1_81212* (three independent transformants) were analyzed using UV-HPLC. Major product 1 was identified as acetyl-tetrahydroxynaphthalene (AT4HN). Minor product 2 was identified as 6,8-dihydroxy-3-methylisocoumarin.

Although many biosynthetic pathways from other fungi have been successfully characterized in Aspergillus oryzae (31, 32), no PKS from the Lecanoromycetes has so far been successfully expressed in this heterologous host for unknown reasons (33). Because the Lobpul1_1267156 BGC is well conserved (Fig. 2), we chose to functionally characterize in A. oryzae the homologous nrPKS from *A. parvulus*, Aspparv1_81212, as it belongs to a complete predicted BGC (Fig. 2B). A. oryzae transformants expressing *Aspparv1_81212* produced two novel compounds compared to transformants carrying an empty vector (Fig. 4 and Fig. S1). Product 1 (retention time [RT] = 9.35 min; maximum UV absorption [UV max] = 197, 230, 278, 322, and 405 nm; *m/z* = 233 [M − H]^−^) exhibits a yellow color and has an exact mass of 235.0577 [M + H]^+^ as determined by high-resolution mass spectrometry (HRMS) (Fig. S2). Product 1 was identified as 2-acetyl-1,3,6,8-tetrahydroxynaphthalene (AT4HN) (Fig. 4) using nuclear magnetic resonance (NMR) (Data Set S5) and by comparing mass and UV spectra to previously published data (34). Product 2 (RT = 8.65 min; UV max = 197, 241, 276, and 325 nm; *m/z* = 191 [M − H]^−^) was identified as the pyrone 6,8-dihydroxy-3-methylisocoumarin based on NMR analyses (Data Set S5) and published data (35). Such pyrones are known shunt metabolites from nrPKSs when the final thioesterase (TE) domain is inactivated (34). However, such shunt pyrones harbor the same length as that of the polyketide released by the fully functional nrPKS (34). While product 1 is a hexaketide released through TE-catalyzed Claisen cyclization, product 2 is a pentaketide that seems to be released after the incorporation of four malonyl-CoAs through spontaneous O-C cyclization (34). Analysis of the transcripts in A. oryzae transformants showed that a single mature nrPKS is expressed (Fig. S1), meaning that this nrPKS can release two polyketides of different lengths. The three transformants yielded AT4HN as the major product (83, 56, and 350 mg/L) compared to the pyrone (7, 13, and 219 mg/mL). The yield difference between the transformants most likely reflects differences in gene expression due to the integration of the plasmid at different genomic loci. These results demonstrate that the *Aspparv1_81212* homologue encodes a PKS that releases the hexaketide AT4HN as the first stable intermediate. Thus, group XI PKSs produce the same backbone as those of certain PKSs in group II (Fig. 1).

10.1128/mbio.00223-22.1 FIG S1 Expression of *Aspparv1_81212* in Aspergillus oryzae transformants. (A) Expression of *Aspparv1_81212* and *H2B* genes in Aspergillus oryzae transformants. M indicates the BenchTop 1-kb ladder (Promega), the negative control contains water, and the positive control for *Aspparv1_81212* contains plasmid pTYGSarg::Aspparv1_81212. (B) Analysis of *Aspparv1_81212* expression by A. oryzae transformants. F1 to F7 designate fragments of the *Aspparv1_81212* gene; M designates the BenchTop 1-kb ladder (Promega). All fragments have the expected sizes (F1, 1,007 bp; F2, 1,059 bp; F3, 1,044 bp; F4, 1,030 bp; F5, 1,035 bp; F6, 1,029 bp; F7, 628 bp). Download FIG S1, PDF file, 0.8 MB. Copyright © 2022 Mosunova et al. 2022 Mosunova et al. https://creativecommons.org/licenses/by/4.0/ This content is distributed under the terms of the Creative Commons Attribution 4.0 International license.

10.1128/mbio.00223-22.2 FIG S2 High-resolution mass spectrometry (HRMS) data for metabolite 1 produced by Aspergillus oryzae NSAR1 transformants expressing *Aspparv1_81212*. The compound with molecular formula C_12_H_11_O_5_ and a mass of 235.0577 (highlighted in green) is consistent with acetyl tetrahydroxynaphthalene. Download FIG S2, PDF file, 0.2 MB. Copyright © 2022 Mosunova et al. 2022 Mosunova et al. https://creativecommons.org/licenses/by/4.0/ This content is distributed under the terms of the Creative Commons Attribution 4.0 International license.

10.1128/mbio.00223-22.10 DATA SET S5 ^1^H and ^13^C NMR spectra of products 1, 2, and 4. Download Data Set S5, PDF file, 0.6 MB. Copyright © 2022 Mosunova et al. 2022 Mosunova et al. https://creativecommons.org/licenses/by/4.0/ This content is distributed under the terms of the Creative Commons Attribution 4.0 International license.

### The group XI biosynthetic gene cluster is linked to the production of 6-*O*-methylasparvenone and ethylparvulenone in Aspergillus parvulus.

**FIG 5:**
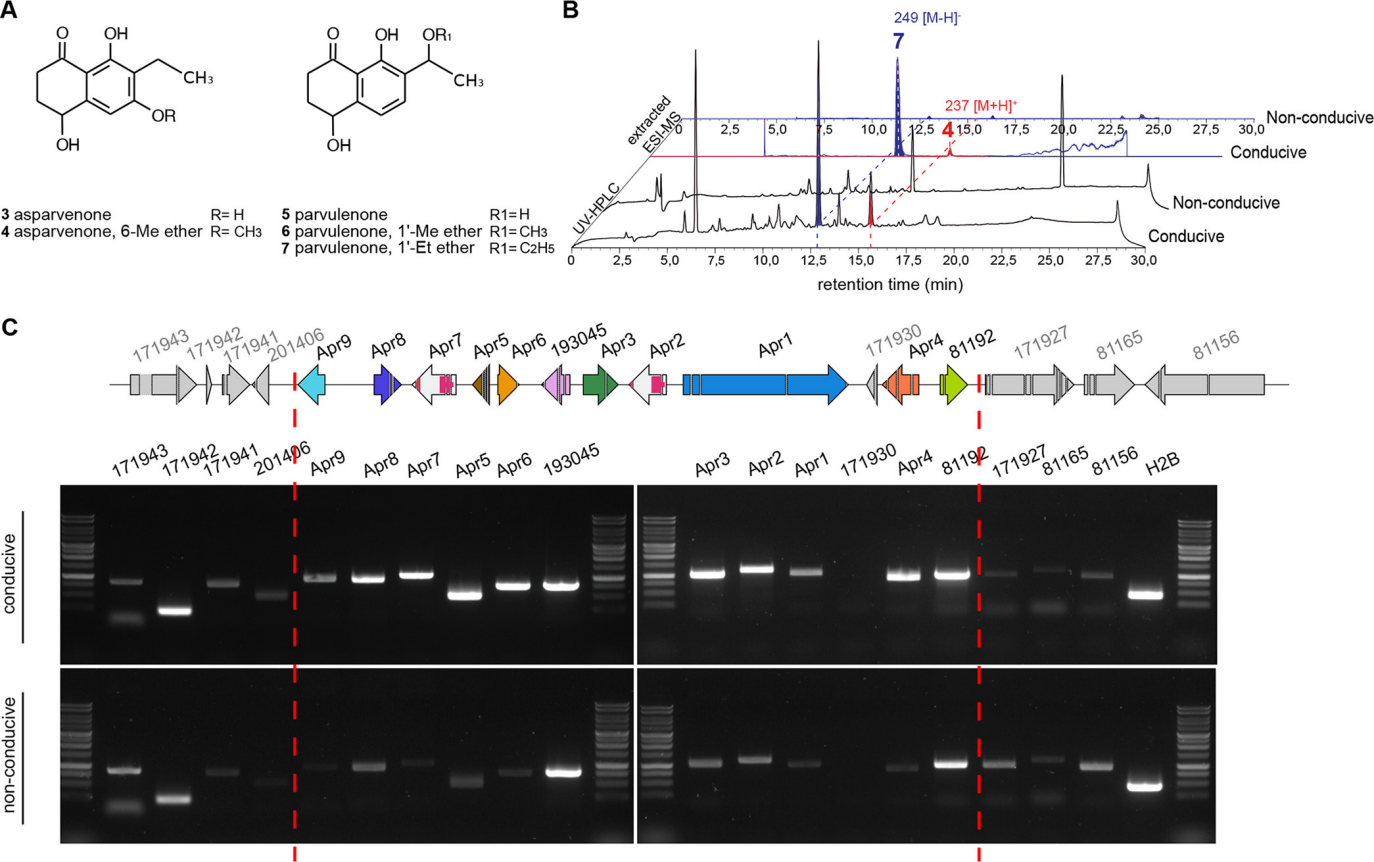
Correlation between the expression of the group XI biosynthetic gene cluster and the production of naphthalenone compounds in Aspergillus parvulus. (A) Chemical structures of naphthalenone compounds reported for *A. parvulus*. (B) UV-HPLC traces of organic extracts obtained from a 6-day-old *A. parvulus* culture in malt extract (conducive) or Czapek-Dox (nonconducive) liquid medium. Analysis of UV spectra and extraction of mass ions (electrospray ionization-mass spectrometry [ESI-MS]) of naphthalenone compounds shown in panel A identified products 4 and 7, provided as representative of results from three replicates. (C) Expression of predicted genes at the *Aspparv1_81212* locus in *A. parvulus* grown under conducive and nonconducive conditions as determined by RT-PCR. The *H2B* housekeeping gene was used as an expression control.

*A. parvulus* is known to produce several polyketides that could structurally derive from compound 1, namely, the naphthalenone asparvenone compound 3, parvulenone compound 5, and methylated or ethylated derivatives 4, 6, and 7 (36) (Fig. 5A). Analysis of organic extracts from *A. parvulus* grown under conditions conducive to naphthalenone production (37) detected the presence of compound 4 (RT = 15.25 min; *m/z* = 237 [M + H]^+^; UV max = 219 and 288 nm) and compound 7 (RT = 12.6 min; *m/z* = 249 [M − H]^−^; UV max = 216, 262, and 308 nm) (Fig. 5B). Product 4 was confirmed to be 6-*O*-methylasparvenone by NMR (Data Set S5).

We then assessed if the predicted *Aspparv1_81212* BGC is expressed when compounds 4 and 7 are produced. With the exception of the predicted and not conserved thioreductase *Aspparv1_171930* gene, all genes predicted to be part of the BGC were found to be significantly expressed compared to the housekeeping gene *H2B* (Fig. 5C). The *H2B* transcript exhibit similar signal intensities in both cultures. In comparison to *H2B* signals, a condition nonconducive to naphthalenone production shows the very limited expression of most genes apart from the genes encoding an NmrA-like protein (*Aspparv1_193045*) and a GroES-like alcohol dehydrogenase (*Aspparv1_81192*), which exhibit similar or slightly lower expression levels. These results make it uncertain whether these two genes belong to the BGC or not. The flanking genes *Aspparv1_171941* and *Aspparv1_201406* seem to be expressed at slightly higher levels under conducive conditions, but they encode a mitochondrial carrier protein and a cupin protein, respectively (Table 1), which are unlikely to be involved in naphthalenone biosynthesis. The other flanking and housekeeping genes do not show coregulation (Fig. 5C). These results show that most of the genes from the predicted BGC in *A. parvulus* are coregulated and that their expression correlates with the production of naphthalenones. Thus, the predicted BGC comprises 9 genes likely involved in the production of asparvenone and derivatives, and the corresponding genes were named *APR1* to *APR9* (Table 1).

**TABLE 1:** Functions of proteins encoded in the predicted group XI biosynthetic gene cluster and flanking genes in Aspergillus parvulus^*a*^

Protein ID	Gene name	Protein function	Pfam domain, E value
171943		Protein kinase	PF00069.27, 4.4e−49
			PF07714.19, 2.3e−23

171942		Unknown	No hit

171941		Mitochondrial carrier protein	PF00153.29, 2.9e−07

201406		Cupin superfamily protein	PF06172.13, 2.0e−51

201405	*APR9*	Zn/Cys transcription factor	PF00172, 7.37e−09

171936	*APR8*	Aldo-keto reductase	PF00248, 6.40e−144

201403	*APR7*	FAD-binding oxidoreductase	PF01565, 7.49e−19
			PF08031, 2.42e−11

201402	*APR5*	Scytalone dehydratase	PF02982, 6.74e−57

213579	*APR6*	T4HN reductase	PF13561, 1.26e−91

193045		NmrA-like protein	PF05368, 4.70e−91

171934	*APR3*	*O*-Acyltransferase	PF13813, 6.04e−20

81217	*APR2*	FAD-binding oxidoreductase	PF01565, 4.34e−29
			PF08031, 4.12e−06

81212	*APR1*	Nonreducing polyketide synthase	SAT (PF16073), 1.04e−57
			KS (PF00109), 1.07e−82
			KS_C (PF02801), 8.67e−41
			AcT (PF00698), 3.56e−38
			PT (TIGR04532), 8.65e−13
			PP-b (PF00550), 21.89e−07
			PP-b (PF00550), 2.06e−10
			TE (PF00975), 3.61e−23

171930		Thioredoxin	PF00085.22, 3.8e−19

193042	*APR4*	*S*-Adenosylmethionine-dependent *O*-methyltransferase	PF00891, 3.87e−20

81192		GroES-like alcohol dehydrogenase	PF08240, 4.76e−07
			PF00107, 6.14e−06

171927		WD domain-containing protein	PF11816.10, 6.4e−59
			PF00400.3, 1.0e−05

81165		PP loop family protein	PF01171.22, 1.6e−48

81156		RhoGEF protein	PF00621.22, 4.1e−25
			PF12015.10, 1.7e−11

a T4HN, tetrahydroxynaphthalene.

## DISCUSSION

### Evolutionary relationships of biosynthetic gene clusters inform fungal chemical diversity.

The phylogenetic dereplication of nrPKSs encoded in the genomes of eight lichenizing fungi has revealed two new phylogenetic groups, of which group XI was not functionally characterized. Comparative genomics allowed the prediction of a BGC that was found to be expressed in *A. parvulus* when naphthalenone compounds were produced. These findings demonstrate that the combination of phylogenetic dereplication and comparative genomics is a powerful strategy that not only provides new insights into the evolution of fungal BGCs but also allows relevant prioritization of functional characterization without *a priori* knowledge about chemical structures.

Although chemical backbone 1 produced by Apr1 is already known (38), this result was unexpected because group XI is a sister clade of the anthraquinone-producing group V nrPKSs and is not related to group IIa, which comprises nrPKSs that are known to produce compound 1 (Fig. 1). Similar to orsellinic acid that is produced by nrPKSs from distantly related clades (Fig. 1), compound 1 appears to be a common chemical backbone produced by enzymes that have diverged long ago.

Compound 1 is a precursor of DHN melanin in certain species like Exophiala dermatitidis, in which it is further converted to T4HN via the removal of the acetyl group by the polyketide-shortening enzyme WdYg1p (38). A similar reaction is catalyzed by the homologue Ayg1 to convert YWA1 into T4HN in A. fumigatus (39). No homologue of WdYg1p could be identified in the *A. parvulus* genome, indicating that compound 1 could not be converted to T4HN in this fungus. Similarly, several homologues of T4HN reductase and scytalone dehydratase were found encoded in the *A. parvulus* genome, but none of them correspond to paralogues involved in the DHN melanin pathway (Fig. 3). Similarly, other Aspergillus species included in this study lack these paralogues (Fig. 3), suggesting that the DHN melanin pathway has been lost in all of them. In contrast, Dothideomycetes and Leotiomycetes species carry both DHN melanin and naphthalenone BGCs, and a few appear to also contain a BGC related to the cladofulvin one. Noteworthy, in *O. maius*, the closest homologues of both the hydroxynaphthalene reductase and scytalone dehydratase genes form an outgroup to the group XI clades (Fig. 3; see also Table S1 in the supplemental material) and are located at another locus next to genes encoding an nrPKS (*Oidma1_51005*) and a cytochrome P450 monooxygenase (*Oidma1_157855*). Consistent with the phylogenetic position, these paralogues correspond to a different biosynthetic pathway in this species. Altogether, the phylogenetic analyses and genomic organizations suggest that the group XI BGC has an ancestral origin, possibly with the recruitment of paralogues from the DHN or cladofulvin pathways after gene duplication and of other tailoring genes that have resulted in the BGC for naphthalenone production in fungi.

### Gene content diversification of the group XI biosynthetic gene cluster correlates with the chemical diversity of naphthalenone compounds.

**FIG 6:**
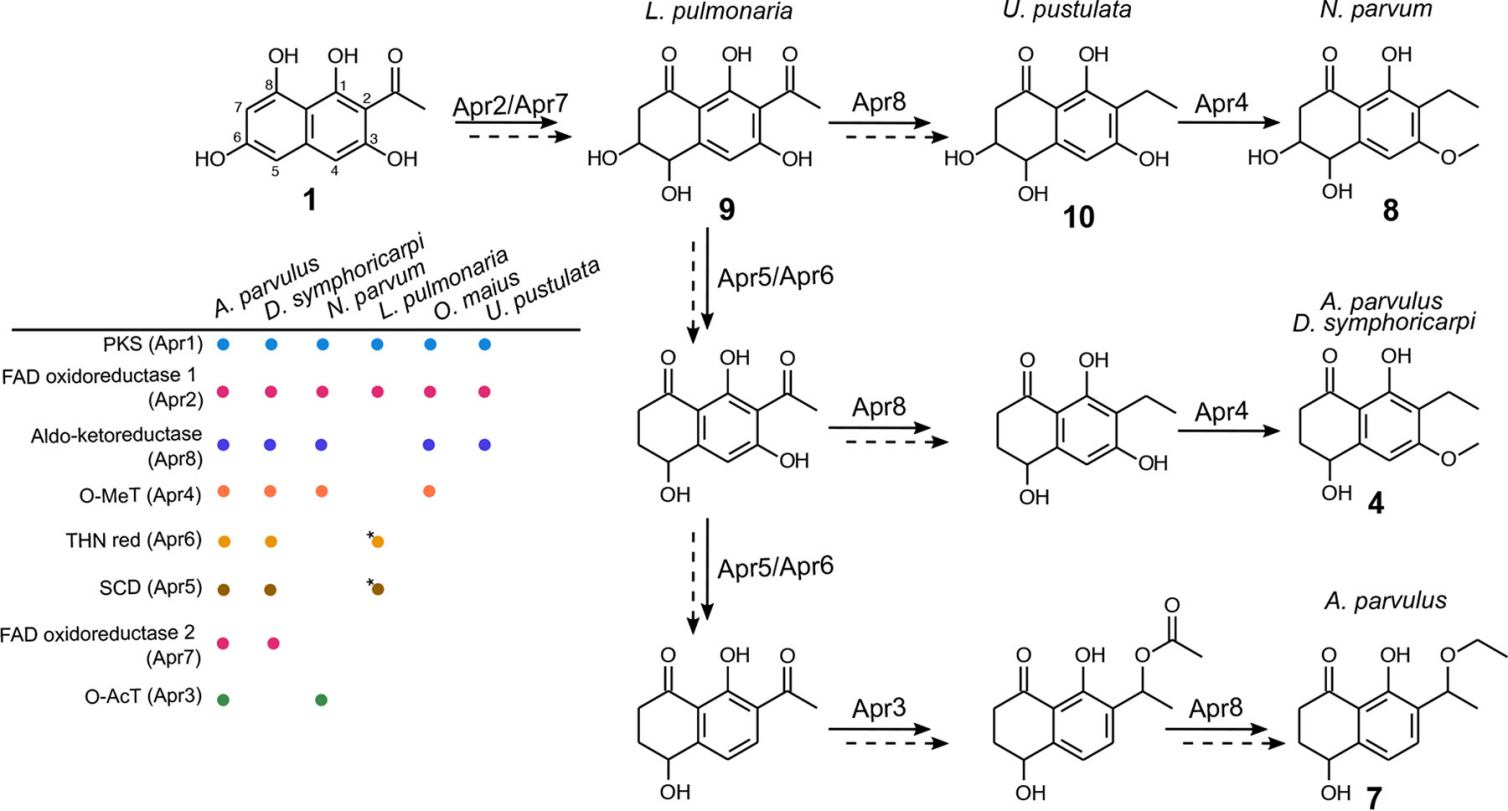
Prediction of enzymatic requirements for the production of diverse naphthalenones in fungi. Aspergillus parvulus produces compounds 4 and 7, while *Neofusicoccum parvum* produces compound 8, consistent with the gene component of the group XI biosynthetic gene cluster (BGC). Based on the BGC content, Apr2 and Apr7 are likely responsible for the hydroxylation at C-5. Apr5 and Apr6 are expected to jointly remove the hydroxyl groups at C-3 and C-6. Apr8 is predicted to reduce and Apr3 is predicted to acetylate the keto group, respectively. The combination of Apr3 and Apr8 is expected to be responsible for yielding compound 7. Apr4 is predicted to be responsible for the methylation of the oxygen at C-3. Dashed arrows indicate that several steps are most likely required between hypothetical intermediates, either spontaneously or through the action of enzymes that remain to be identified. The predicted BGC in *Lobaria pulmonaria* suggests that this species produces the hypothetical compound 9, while *Umbilicaria pustulata* and *Oidiodendron maius* are predicted to produce derivatives of the hypothetical compound 10. Genes with identified disruptive mutations are labeled with a star. *O*-MeT, *O*-methyltransferase; red, reductase; O-AcT, *O*-acetyltransferase; SCD, scytalone dehydratase.

Our results suggest that product 1 is the initial chemical backbone to produce products 4 and 7, which is consistent with the previous proposition that a hexaketide precursor is the starting molecule of product 4 (36). We also found that the pentaketide pyrone product 2 is released at the same time as product 1. Traces of product 2 were detected in organic extracts of *A. parvulus* (Fig. S3), which indicates that the release of product 2 is likely not a misfunction of the nrPKS in A. oryzae. However, the release of product 2 in *A. parvulus* needs to be confirmed to validate this hypothesis. In addition, the predicted catalytic activities of tailoring enzymes encoded at the *APR1* locus are consistent with necessary modifications to convert product 1 into product 4 or 7. DHN biosynthesis in fungi involves the removal of two hydroxyl groups through the sequential action of a hydroxynaphthalene reductase (4Hnr/Arp2 and 3Hnr) and a scytalone dehydratase (Scd1/Arp1) (26). Similarly, ClaB and ClaC in C. fulvum are paralogues of 3Hnr and Scd1, which catalyze the removal of a hydroxyl group from emodin to yield chrysophanol hydroquinone in the cladofulvin pathway (27). In *A. parvulus*, the removal of the C-3 and C-6 hydroxyl groups, as found in products 4 and 7 compared to product 1, likely involves Apr5 and Apr6, the paralogues of Arp1 and Arp2, respectively (Fig. 3 and 6). The presence of the predicted *O*-methyltransferase (Apr4) and *O*-acyltransferase (Apr3) encoded in the BGC is consistent with the methylation and ethylation observed in products 4 and 7, respectively (Fig. 6). A particular feature of product 4 is the reduction of the ketone, which could involve Apr8, a predicted aldo-keto reductase (Fig. 6), and possibly another enzyme to remove the resulting hydroxyl group. In a similar reaction to produce product 4, the acetyl group added by Apr3 is likely reduced by Apr8, followed by the removal of the hydroxyl group to yield the ethyl group. The oxidoreductases Apr2 and Apr7 could be involved in the complete reduction of the aromatic ring and/or the selective oxidation of C-5 (Fig. 6). Because the BGC is fully conserved in all Aspergillus species in which an *APR1* orthologue was found as well as in Atropellis piniphila, Sporormia fimetaria, Dothidotthia symphoricarpi, Karstenula rhodostoma, Paraconiothyrium sporulosum, *Bimuria novae-zelandiae*, and D. sadasivanii (Fig. 2B and Table S1), we expect all these species to produce naphthalenone molecules related to products 4 and 7. The absence of *APR3* in D. symphoricarpi indicates that derivatives of product 4 may be produced only (Fig. 2B and Table S1).

10.1128/mbio.00223-22.3 FIG S3 Search for product 2 in organic extracts from Aspergillus parvulus. Shown is the extracted mass of product 2 (191 in negative mode) in extracts under conducive and nonconducive conditions, with UV spectra. Purified product 2 produced by an Aspergillus oryzae transformant is shown as a reference. Download FIG S3, PDF file, 0.2 MB. Copyright © 2022 Mosunova et al. 2022 Mosunova et al. https://creativecommons.org/licenses/by/4.0/ This content is distributed under the terms of the Creative Commons Attribution 4.0 International license.

The fungus *N. parvum* is known to produce diverse naphthalenones, including botryosphaerone D compound 8 (40), which differs from compound 4 only by the presence of the C-6 hydroxyl group (Fig. 6). The group XI BGC in *N. parvum* comprises five genes only (Fig. 2), including *APR8* and *APR4*, which encode the predicted aldo-keto reductase and *O*-methyltransferase, consistent with the chemical structure of compound 8. The presence of the *O*-acyltransferase Apr3, although encoded at a different locus in the genome (Fig. 2B), suggests that *N. parvum* could also produce a molecule related to compound 7. Orthologues of *APR5* and *APR6* could not be identified in the genome of *N. parvum*, which likely explains why both the C-3 and C-8 hydroxyl groups remain in compound 8 (Fig. 3 and Table S1). Because the *N. parvum* genome also lacks the oxidoreductase gene *APR7* (Table S1), this suggests that both Apr2 and Apr7 may be functionally redundant. Further functional validation of these tailoring enzymes is being performed to address such biosynthetic hypotheses.

The BGC composition in *L. pulmonaria* and Melanops tulasnei is limited to *APR1* and *APR2* only, as in *L. pulmonaria*, both the *APR5* and *APR6* genes contain disruptive mutations that make the proteins likely not functional (Table S1 and Data Sets S1 to S3). Thus, these two species could produce compounds related to compound 9 (Fig. 6). In addition to *APR1* and *APR2*, *U. pustulata* and *O. maius* also comprise the aldo-keto reductase *APR8* gene (Fig. 2B and Table S1), suggesting that they might produce compounds related to compound 10 (Fig. 6). The presence of the *O*-methyltransferase *APR4* gene in *O. maius* suggests that a methylated derivative of compound 10 is produced (Fig. 6). Assigning functions of tailoring genes to biosynthetic steps of the naphthalenone pathway in *A. parvulus* will allow the reconstruction of pathways encoded in the genomes of other fungi, including the lichen mycobionts, and validate these hypotheses.

### Conclusions.

Combining phylogenetic dereplication and comparative genomics is a powerful strategy to prioritize the functional characterization of BGCs without any *a priori* knowledge other than evolutionary novelty. While most fungal genomes are used to either list their production potential or find BGCs for known molecules of interest, our approach generates knowledge on the evolution of fungal BGCs, potentially finds novel molecules, and otherwise links genes to already known molecules, as found here. This strategy is very promising to unlock the full rational exploitation of fungal genomes for BGC characterization and molecule discovery.

## MATERIALS AND METHODS

### Fungal genomes and sequences.

Genome assemblies and gene predictions of *Cladonia grayi* Cgr/DA2myc/ss v2.0, *Xanthoria parietina* 46-1-SA22 v1.1, *Usnea florida* ATCC 18376 v1.0, *Lobaria pulmonaria* Scotland reference genome v1.0, *Acarospora strigata* CBS 132363, *Dibaeis baeomyces*, *Graphis scripta* CBS 132367, and *Umbilicaria pustulata* were retrieved from the Joint Genome Institute (JGI) MycoCosm repository (41). BGCs were predicted using fungiSMASH 4 with default parameters (42). Predicted nrPKSs were selected based on the presence of the signature SAT starter unit:ACP transacylase (PF16073) and PT product template (TIGR04532) conserved domains. Gene models for homologues of *APR1*, *APR5*, *APR6*, and *APR8* were curated manually (see Data Set S4 in the supplemental material). Characterized nrPKSs were retrieved from the Minimum Information about a Biosynthetic Gene Cluster (MIBiG) database (24) and from the literature (Data Sets S1 to S3). Close homologues of *A. parvulus* Apr1 were retrieved from the MycoCosm repository using BLASTp. Another BLASTp search with each *A. parvulus* protein predicted in the pathway was performed on filtered proteins of each species containing the group XI BGC, allowing the identification of genes located at a different locus (Table S1).

### Phylogenetic trees.

Protein alignments were performed using Clustal Omega v1.2.4 (43), with the KS keto-synthase domain PF00195.19 as a guide (parameters –hmm –in) in the case of nrPKS sequences. Poorly aligned regions were removed using trimaL 1.4.rev15 (build 2013-12-17; parameter -automated1) (44). Maximum likelihood trees were built with IQ-TREE v1.6.11-he860b03_0 bioconda (45) with model finder (30) and ultrafast bootstrapping as well as an approximate Bayes test (46) and a Shimodaira-Hasegawa approximate likelihood-ratio test (47) (parameters –bb 1000 -nt AUTO -mset LG -alrt 1000 -abayes -m MFP). The resulting trees were visualized using iTOL (48). All curated alignments and phylogenetic tree files are provided in Data Sets S1 to S3.

### Fungal strains and growth conditions.

*A. parvulus* CBS 136.61 from the CBS collection (Westerdijk Fungal Biodiversity Institute, The Netherlands) was grown for 6 days in malt extract broth (MB) (filtered malt extract at 400 mL/L [pH 7.0]) liquid medium at 25°C under constant agitation at 200 rpm or on malt extract agar (MEA) (50 g/L [pH 5.4]; Oxoid) plates. For total RNA isolation and secondary-metabolite extraction, *A. parvulus* was grown for 6 days in 50 mL Difco Czapek-Dox (CZD) broth (BD, Franklin Lakes, NJ) or MB (pH 3.5) liquid medium at 25°C under constant agitation at 200 rpm. A. oryzae NSAR1 was grown on MEA plates for 5 days at 30°C. A. oryzae transformants were grown on selective CZD medium without arginine (35 g/L Difco CZD broth [BD, Franklin Lakes, NJ], 1 g/L ammonium sulfate [Sigma-Aldrich, St. Louis, MO], 0.5 g/L adenine [Sigma-Aldrich, St. Louis, MO], 1.5 g/L methionine [Sigma-Aldrich, St. Louis, MO]). For the induction of the P*amyB* promoter and polyketide production (49), transformants were grown at 30°C for 5 days in Yeast Malt Agar (YMA) (3 g/L Difco yeast extract, 3 g/L Difco malt extract, 5 g/L Difco Bacto peptone, 10 g/L glucose [Merck, Kenilworth, NJ]) liquid or agar (Ferwo 700 agar) medium. The mycelium from transformants grown in liquid YMA cultures was used to extract total RNA.

### Nucleic acid extraction and RT-PCR.

The mycelium of *A. parvulus* or A. oryzae from liquid cultures was filtered through a paper filter, frozen in liquid nitrogen, and ground using a mortar and pestle. Genomic DNA was isolated using the DNeasy plant minikit (Qiagen, Hilden, Germany) according to the manufacturer’s recommendations. For total RNA extraction, 100 mg of the ground mycelium was mixed with 1 mL of Invitrogen TRIzol reagent (Thermo Fisher Scientific, Waltham, MA) in a 1.5-mL microcentrifuge tube and incubated for 5 min at 25°C. The resulting lysate was mixed with 0.2 mL chloroform, gently mixed by hand, and incubated for 5 min. Samples were centrifuged at 12,000 × *g* for 15 min at room temperature. The aqueous phase was transferred into a new microcentrifuge tube, mixed with 0.5 volumes of 100% ethanol, and loaded into a column from the NucleoSpin RNA extraction kit (Macherey-Nagel, Allentown, PA). Downstream steps were performed according to the manufacturer’s protocol. Five hundred nanograms of total RNA was used to synthesize cDNA using oligo(dT) primers and GoScript reverse transcription (RT) mix (Promega, Madison, WI) according to the manufacturer’s protocol. PCR was performed for each *A. parvulus* gene at the *APR1* locus and the housekeeping control gene *H2B* (Table S2) using GoTaq DNA polymerase (Promega, Madison, WI). To confirm *APR1* expression in A. oryzae transformants, primers specific for the *A. parvulus APR1* and A. oryzae *H2B* genes (Table S2) were used with GoTaq DNA polymerase (Promega, Madison, WI).

10.1128/mbio.00223-22.5 TABLE S2 Oligonucleotides used in this study. Download Table S2, XLSX file, 0.01 MB. Copyright © 2022 Mosunova et al. 2022 Mosunova et al. https://creativecommons.org/licenses/by/4.0/ This content is distributed under the terms of the Creative Commons Attribution 4.0 International license.

### Gene amplification and plasmid digestion.

The five exons of *APR1* (JGI protein identifier 81212) were amplified from genomic DNA of *A. parvulus* using primers that harbor 15-bp sequences homologous to the previous and next exons (Table S2), with the exception of the forward and reverse primers used to amplify the first and last exons, respectively, which harbor 30-bp sequences homologous to the pEYA2 plasmid (49) (Table S2). The last exon was amplified to include a 100-bp downstream terminator sequence. All PCR fragments were amplified using Phusion high-fidelity DNA polymerase (Thermo Fisher Scientific, Waltham, MA) according to the manufacturer’s protocol. One microgram of the pEYA2 plasmid was digested overnight with 10 U NotI at 37°C (Promega, Madison, WI). Fragments of the expected size and the linearized plasmid were purified from a 0.8% agarose gel or directly from the PCR mix using a Geneclean II kit (MP Biomedicals, Santa Ana, CA).

### Transformation-associated recombination in Saccharomyces cerevisiae.

A strain of Saccharomyces cerevisiae BMA 64 with a *ura3*^−^ auxotrophic marker was used for transformation-associated recombination according to a protocol adapted from the one described previously (50). S. cerevisiae was grown overnight at 30°C in 3 mL yeast extract-peptone-dextrose (YPD) medium (20 g/L d-glucose [Sigma-Aldrich, St. Louis, MO], 20 g/L Difco peptone, 10 g/L Difco yeast extract). Two milliliters containing 10^8^ cells was transferred into 50 mL YPD medium and incubated at 30°C under agitation at 200 rpm for about 5 h until reaching an optical density at 600 nm (OD_600_) of 1 to 1.5. Yeast cells were centrifuged for 5 min at 2,500 rpm at 4°C. The cells were resuspended in 20 mL of a filter-sterilized lithium acetate (LiAc)-dithiothreitol (DTT) solution (100 mM LiAc, 10 mM DTT, 0.6 M sorbitol, 10 mM Tris-HCl [pH 7.5]) and incubated at room temperature for 30 min under agitation at 100 rpm. The cells were centrifuged for 5 min at 2,500 rpm at 4°C, and the supernatant was discarded. Cells were washed in 7 mL of ice-cold 1 M sorbitol and centrifuged at 2,500 rpm three times. Finally, the cells were resuspended in 400 μL of ice-cold 1 M sorbitol. Eight microliters of the cell suspension was mixed with 6 μL of the NotI-linearized pEYA2 plasmid and 2 μL of each *APR1* exon fragment in a prechilled electroporation cuvette. After a 5-min incubation on ice, cells were electroporated (1,500 V and 200 Ω) and mixed immediately with 1 mL of ice-cold 1 M sorbitol. Cells were incubated for 1.5 h at 30°C without agitation and then centrifuged, and 200 μL was plated onto synthetic dropout agar medium (SDM) (20 g/L agar, 20 g/L d-glucose [Sigma-Aldrich, St. Louis, MO], 1.92 g/L yeast dropout supplements without uracil [Sigma-Aldrich, St. Louis, MO], 6.7 g/L yeast nitrogen base without amino acids [Sigma-Aldrich, St. Louis, MO]). Plates were incubated for 3 to 7 days at 30°C. Yeast transformants were transferred to a new selective plate and grown overnight. Single colonies were transferred into a microcentrifuge tube in 30 μL of 25 mM NaOH and boiled for 10 min at 100°C. Next, 1 μL was used for PCR screening with GoTaq DNA polymerase (Promega, Madison, WI) and primers APR1_F and APR1_R (Table S2). Positive transformants were grown overnight in liquid SDM to isolate the pEYA2::*APR1* plasmid using the Zymoprep yeast plasmid miniprep kit (Zymo Research, Irvine, CA), and the obtained plasmid was subsequently introduced into electrocompetent Escherichia coli DH5α cells (Thermo Fisher Scientific, Waltham, MA) using an electroporation method according to the manufacturer’s protocol. PCR screening was performed by transferring individual colonies into the PCR mixture with GoTaq DNA polymerase (Promega, Madison, WI). The pEYA2::*APR1* plasmid was isolated from confirmed positive clones using the Zyppy plasmid miniprep kit (Zymo Research, Irvine, CA), and the plasmid was validated by sequencing (Macrogen, Seoul, South Korea).

### Construction of the expression vector.

Seventy nanograms of the pEYA2::*APR1* entry vector and 100 ng of the pTYGSarg destination vector (49) were mixed with 1 μL of the Gateway LR Clonase II enzyme (Thermo Fisher Scientific, Waltham, MA) in a 5-μL final volume, and the reaction mixture was incubated at 25°C for 2 h. The total reaction mixture was introduced into chemically competent E. coli DH5α cells (Thermo Fisher Scientific, Waltham, MA) using a heat shock protocol. The pTYGSarg::*APR1* expression vector was isolated from positive colonies using the Zyppy plasmid miniprep kit (Zymo Research, Irvine, CA).

### Transformation A. oryzae NSAR1.

Spores from A. oryzae NSAR1 were harvested from MEA plates in 5 mL of sterile water, and 1 mL of this spore suspension was inoculated into 50 mL of MB liquid medium and grown overnight at 28°C with shaking at 200 rpm. Germinating spores were collected by centrifugation at room temperature for 10 min at 3,500 rpm and resuspended in 25 mL of 0.8 M NaCl. After centrifugation for 10 min at 3,500 rpm at room temperature, germinated spores were resuspended in 10 mL of a freshly made filter-sterilized protoplasting solution (200 mg *Trichoderma* lysing enzyme [Thermo Fisher Scientific, Waltham, MA] and 50 mg Driselase [Thermo Fisher Scientific, Waltham, MA] in 0.8 M NaCl) and incubated at 30°C for 2 to 2.5 h with shaking at 100 rpm. Protoplasts were filtered through sterile Miracloth and then centrifuged for 5 min at 3,000 rpm at 4°C. Protoplasts were resuspended in 200 μL of solution 1 (0.8 M NaCl, 10 mM CaCl_2_, and 50 mM Tris-HCl [pH 7.5]) and aliquoted to 100 μL in 2-mL microcentrifuge tubes. Ten micrograms of the pTYGSarg::*APR1* expression plasmid or the empty vector pTYGSarg was added to protoplasts, and the mixture was incubated on ice for 2 min. One milliliter of solution 2 (60% [wt/vol] polyethylene glycol 3350 [PEG 3350], 0.8 M NaCl, 10 mM CaCl_2_, and 50 mM Tris-HCl [pH 7.5]) was added, and the tubes were gently inverted before incubation at room temperature for 20 min. Protoplasts were then mixed with 25 mL of cooled selective CZD top 1.5% agar without arginine supplemented with 1 M sorbitol and immediately plated onto selective CZD bottom 0.8% agar without arginine supplemented with 1 M sorbitol. Transformation plates were incubated at 30°C for 3 to 10 days.

### Secondary-metabolite extraction and HPLC-MS analyses.

Secondary metabolites from 6-day-old *A. parvulus* or 5-day-old A. oryzae transformant liquid culture filtrates were isolated with a 1:1 volume of ethyl acetate (VWR Chemicals, Radnor, PA). After shaking on an orbital shaker for at least 1 h, the organic phase was transferred to a 50-mL tube and evaporated under nitrogen flow. The resulting solid was dissolved in acetonitrile. Organic extracts were analyzed with a Shimadzu LC-2030 3D-Prominence-i PDA system coupled to a Shimadzu LCMS-2020 mass spectrometer and equipped with a Shimadzu Shim-pack GIST C_18_-HP reversed-phase column (3 μm, 4.6 by 100 mm). The following method was used: a linear gradient of buffer B (0 to 95%) for 20 min, 5 min of 95% buffer B, and then 100% buffer A for 5 min. Water with 0.1% trifluoroacetic acid (TFA) for high-performance liquid chromatography (HPLC) or 0.05% formic acid for mass spectrometry (MS)-coupled analyses was used as buffer A, and acetonitrile (LCMS grade) with 0.1% TFA for HPLC or 0.05% formic acid for MS-coupled analyses was used as buffer B. The flow rate was 1 mL/min or 0.5 mL/min for HPLC or MS-coupled analyses, respectively. The equipment was controlled and results were analyzed using Shimadzu LabSolutions LCMS software.

### Compound purification.

Crude organic extracts were fractionated using a Shimadzu preparative HPLC system consisting of a CBM-20A controller, an LC-20AP pump, an SPD-20A detector, and an FRC-10A fraction collector, equipped with a C_18_ reversed-phase Reprosil column (10 μm, 120 Å, 250 by 22 mm). The system was controlled with Shimadzu LabSolutions software. A 12.5-mL flow was used with a linear gradient of buffer B (0 to 95%), 5 min of 95% buffer B, and then 100% buffer A for 5 min.

### HRMS and NMR.

HRMS was performed using an LCT instrument (Micromass Ltd., Manchester, UK). Calibration was done with sodium formate, and measurements were acquired for samples mixed with sodium formate. ^1^H (600 MHz) and ^13^C (151 MHz) NMR analyses were performed for samples reconstituted in dimethyl sulfoxide (DMSO) on a Bruker 600 spectrometer and analyzed using MNOVA software. Chemical shifts for protons are reported in parts per million downfield from tetramethylsilane and are referenced to residual protium in the solvent (^1^H NMR, DMSO-d_6_ at 2.50 ppm). Chemical shifts for carbons are reported in parts per million downfield from tetramethylsilane and are referenced to the carbon resonances of the residual solvent peak (^13^C NMR, DMSO-d_6_ at 39.52 ± 0.06 ppm). NMR data are represented as follows: chemical shift, multiplicity (s, singlet; bs, broad singlet; d, doublet; dd, doublet of doublet; t, triplet; q, quartet; ddd, doublet of doublet of doublets; dtd, doublet of triplet of doublets; m, multiplet), coupling constants (hertz), and integration.
