# Supplementary material for: Evolution-Informed Discovery of the Naphthalenone Biosynthetic Pathway in Fungi
Source: mBio. 2022 May 26;13(3):e00223-22. doi: 10.1128/mbio.00223-22 (PMC9239057; doi:10.1128/mbio.00223-22)
Supplement: FIG S1 [file mbio.00223-22-s0006.pdf]

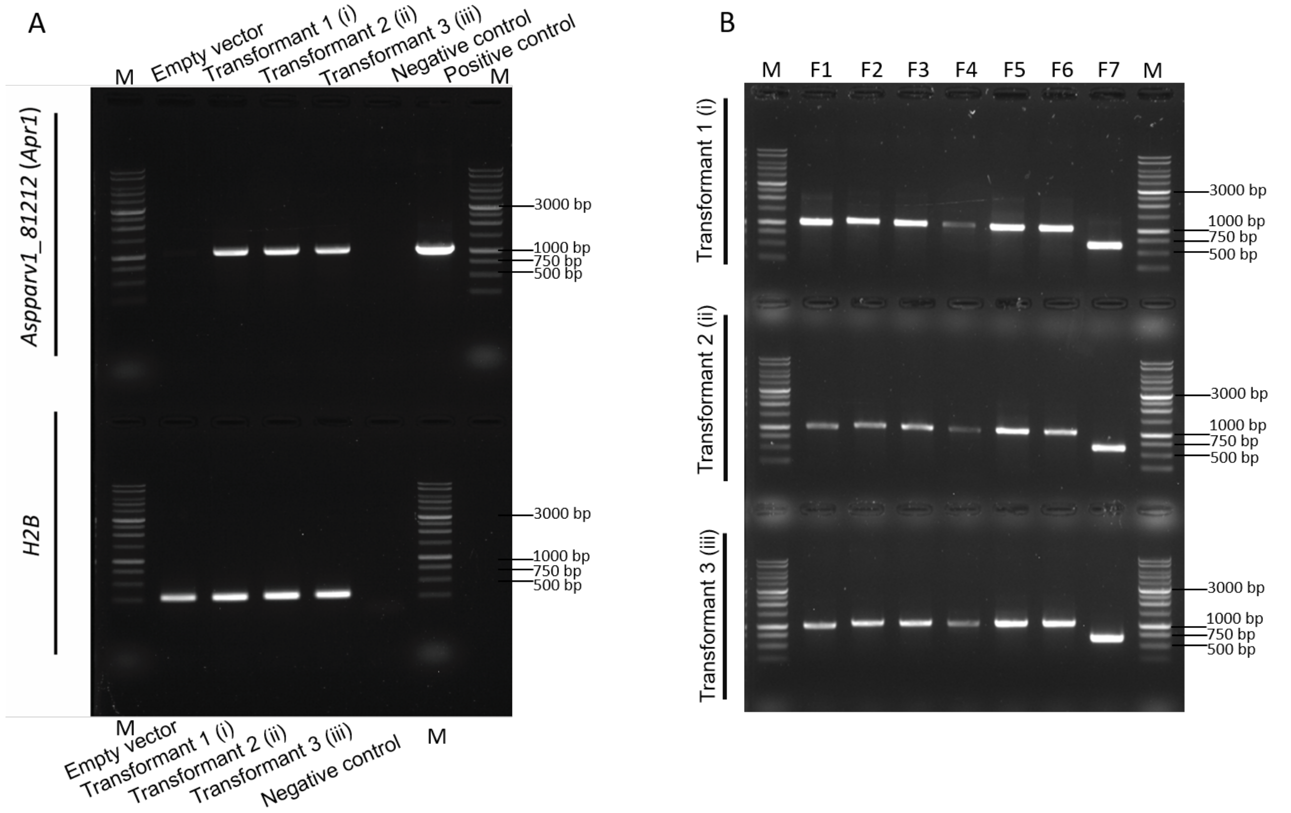

Figure S1. Analysis of expression of *Aspparv1_81212* in *Aspergillus oryzae* transformants. (A) Expression of *Aspparv1_81212* and *H2B* genes in *Aspergillus oryzae* transformants. M indicates BenchTop 1 kb ladder (Promega), negative control contains water, and positive control for *Aspparv1_81212* contains plasmid pTYGSarg::Aspparv1_81212. (B) Analysis of *Aspparv1_81212* expression by *A. oryzae* transformants. F1-F7 designate fragments of *Aspparv1_81212* gene; M designates Benchtop 1kb ladder (Promega). All fragments have expected size (F1 = 1007bp, F2 = 1059bp, F3 = 1044bp, F4 = 1030bp, F5 = 1035bp, F6 = 1029bp, F7 = 628bp).
