# Supplementary material for: Evolution-Informed Discovery of the Naphthalenone Biosynthetic Pathway in Fungi
Source: mBio. 2022 May 26;13(3):e00223-22. doi: 10.1128/mbio.00223-22 (PMC9239057; doi:10.1128/mbio.00223-22)
Supplement: FIG S2 [file mbio.00223-22-s0007.pdf]

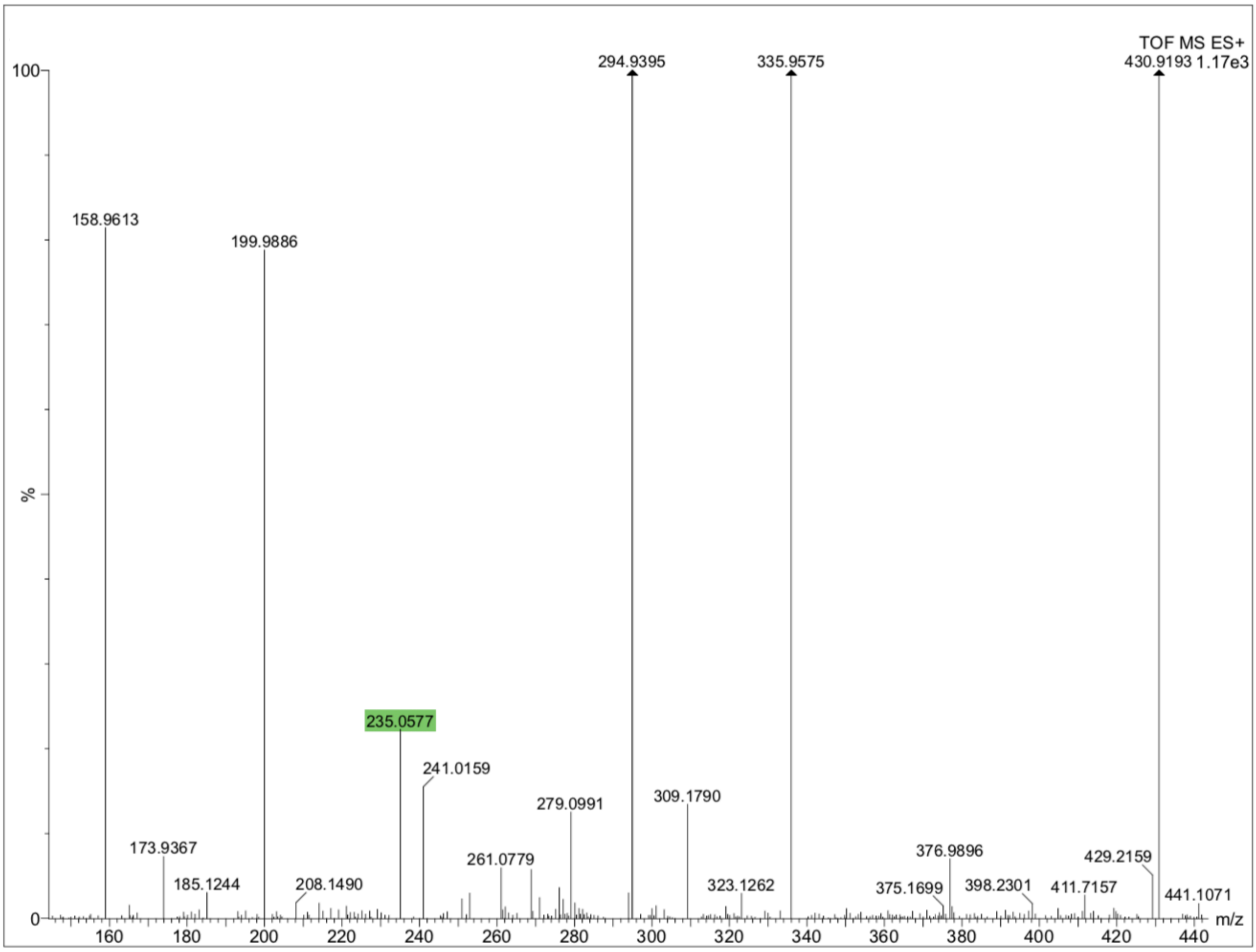

Single Mass Analysis
Tolerance = 10.0 mDa / DBE: min = -1.5, max = 50.0
Isotope cluster parameters: Separation = 1.0 Abundance = 1.0%

Monoisotopic Mass, Even Electron Ions
147 formula(e) evaluated with 5 results within limits (up to 50 closest results for each mass)

Minimum:				-1.5		
Maximum:		10.0	5.0	50.0		
Mass	Calc. Mass	mDa	PPM	DBE	Score	Formula
235.0577	235.0580	-0.3	-1.1	8.5	1	C8 H7 N6 O3
	235.0548	2.9	12.4	16.5	5	C19 H7
	235.0606	-2.9	-12.5	7.5	2	C12 H11 O5
	235.0620	-4.3	-18.2	12.5	3	C13 H7 N4 O
	235.0508	6.9	29.6	12.5	4	C14 H7 N2 O2

Figure S2. High Resolution Mass Spectrometry (HRMS) data for metabolite 1 produced by *Aspergillus oryzae* NSAR1 transformants expressing *Aspparv1_81212*. Compound with molecular formula $C_{12}H_{11}O_5$ and mass 235.0577 (highlighted in green) is consistent with acetyl tetrahydroxynaphthalene.
