# Supplementary material for: Evolution-Informed Discovery of the Naphthalenone Biosynthetic Pathway in Fungi
Source: mBio. 2022 May 26;13(3):e00223-22. doi: 10.1128/mbio.00223-22 (PMC9239057; doi:10.1128/mbio.00223-22)
Supplement: DATA SET S5 [file mbio.00223-22-s0002.pdf]

Product 1: ATHN

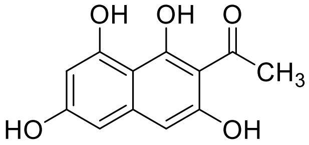

^{1}H NMR (600 MHz, DMSO)

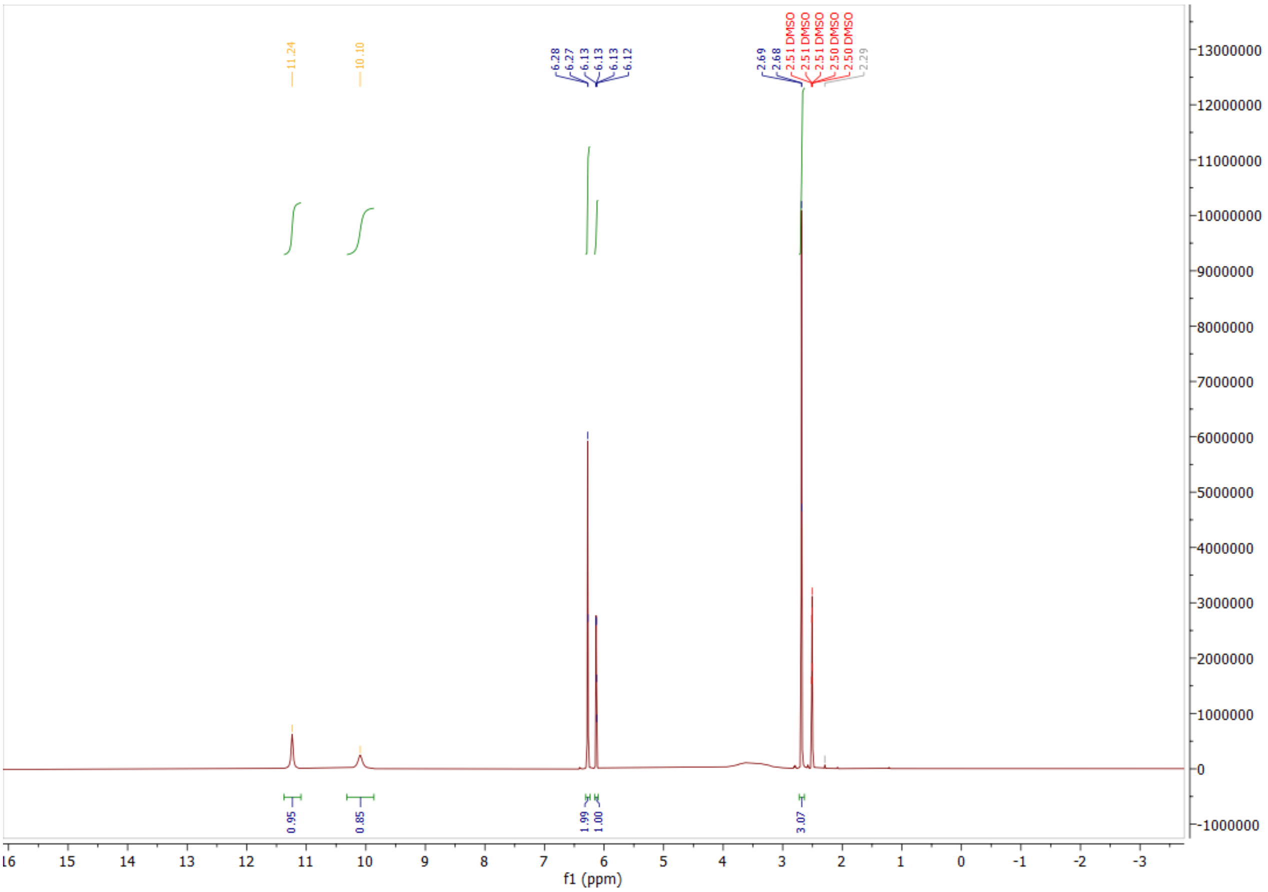

^{1}H NMR (600 MHz, DMSO) δ 11.24 (s, *OH* x2), 10.10 (s, *OH* x2), 6.27 (d, J = 4.0 Hz, 2H), 6.13 (dd, J = 4.2, 2.1 Hz, 1H), 2.68 (s, J = 3.9 Hz, 3H, $COCH_3$).

^{13}C NMR (151 MHz, DMSO)

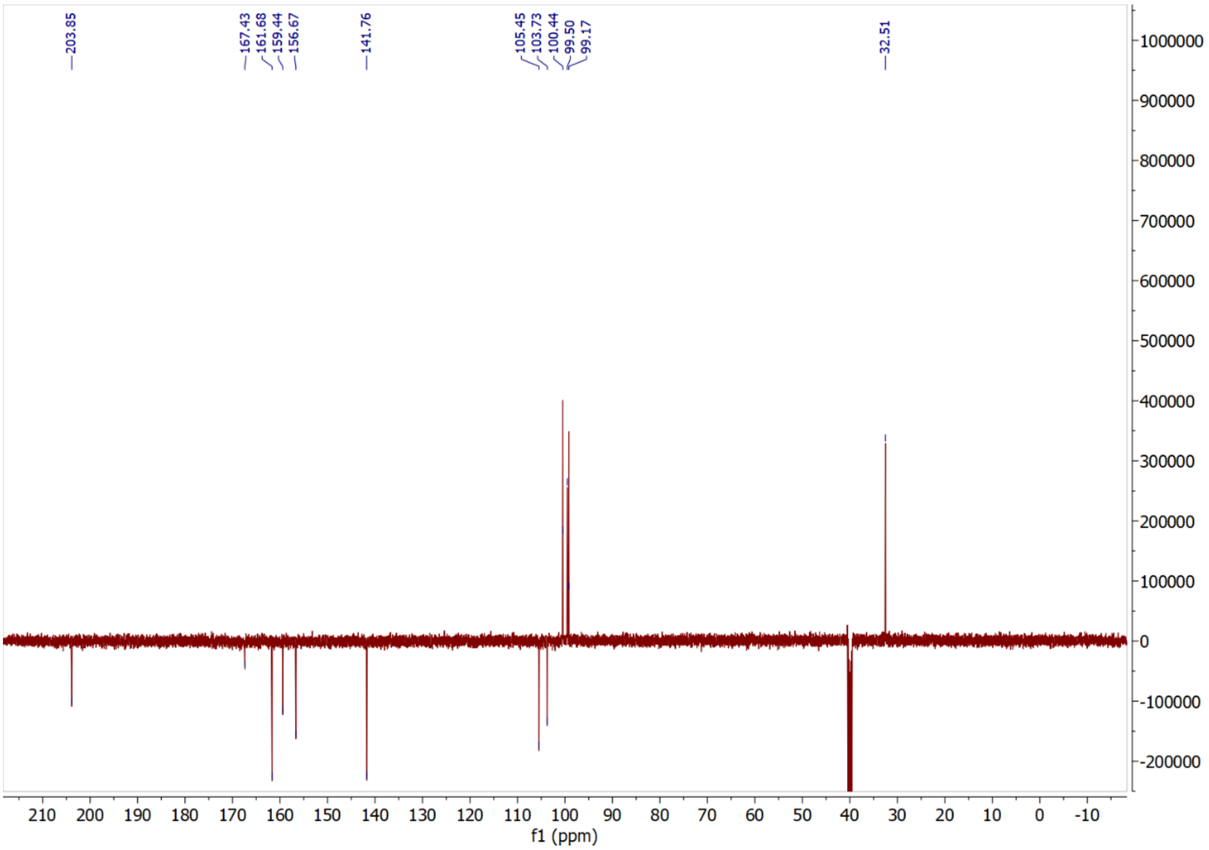

^{13}C NMR (151 MHz, DMSO) δ 203.85 (*C*O-CH_3), 167.43 (*C*-OH), 161.68 (*C*-OH), 159.44 (*C*-OH), 156.67 (*C*-OH), 141.76, 105.45, 103.73, 100.44, 99.50, 99.17, 32.51 (CO*C*H_3).

Product 2: 6,8-dihydroxy-3-methylisocoumarin

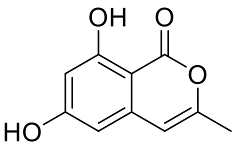

1H NMR (600 MHz, DMSO)

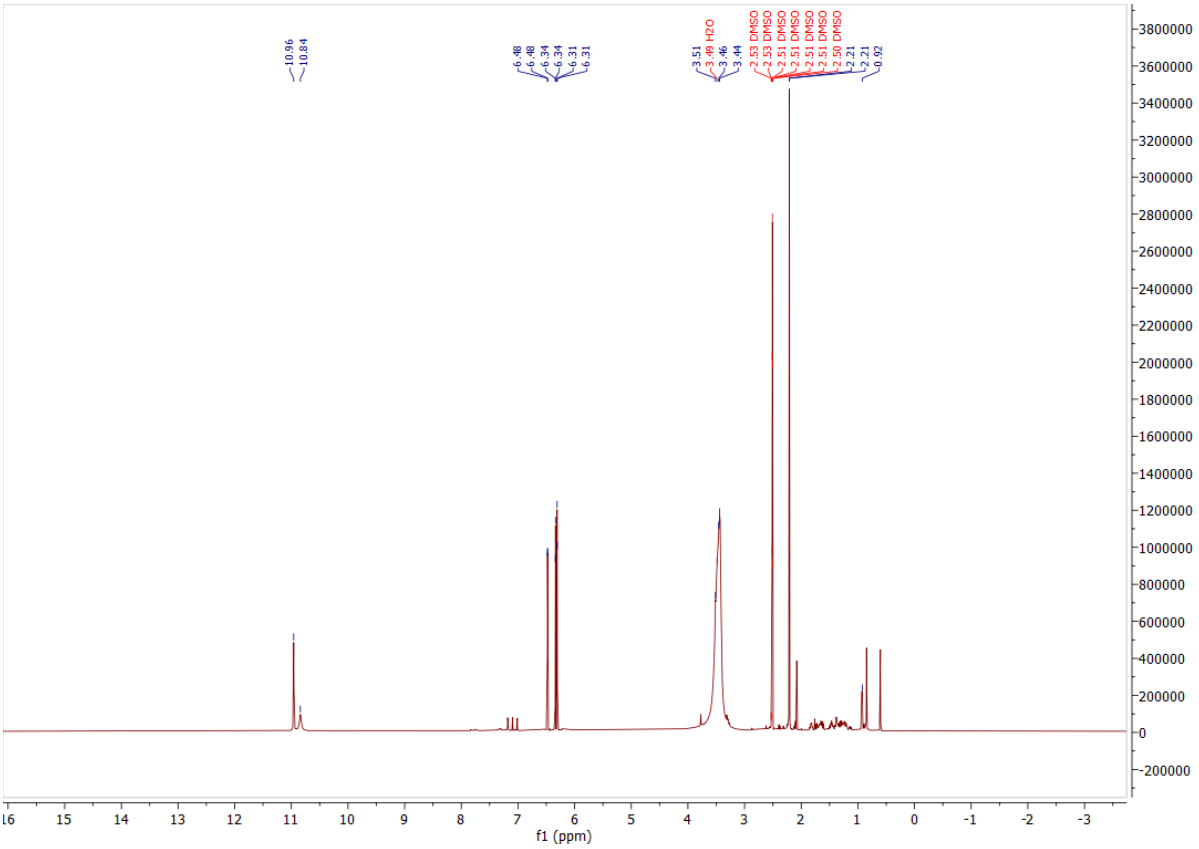

^{1}H NMR (600 MHz, DMSO) δ 10.96 (s, 1H, O*H*), 10.84 (s, 1H, O*H*), 6.48 (d, J = 1.2 Hz, 1H), 6.34 (d, J = 2.2 Hz, 1H), 6.31 (d, J = 2.2 Hz, 1H), 2.21 (d, J = 1.0 Hz, 3H, C*H*$_3$).

^{13}C NMR (151 MHz, DMSO)

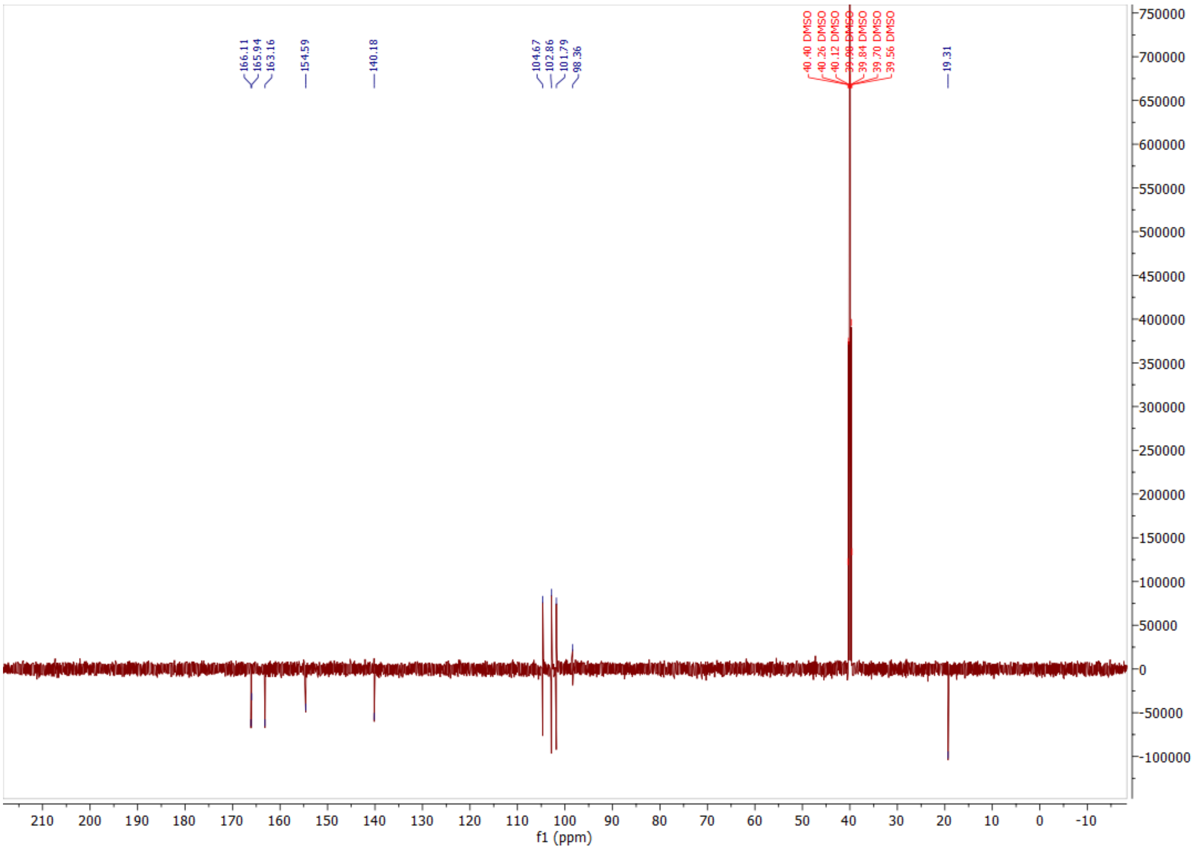

^{13}C NMR (151 MHz, DMSO) δ 166.11 (*C*O, carbonyl), 165.94 (*C*-OH, aromatic), 163.16 (*C*-OH, aromatic), 154.59 (*C*-CH_3), 140.18, 104.67, 102.86, 101.79(*C*H, aromatic), 98.36 (*C*H, aromatic), 19.31 (*C*H_3).

HSQC (600 MHz, DMSO)

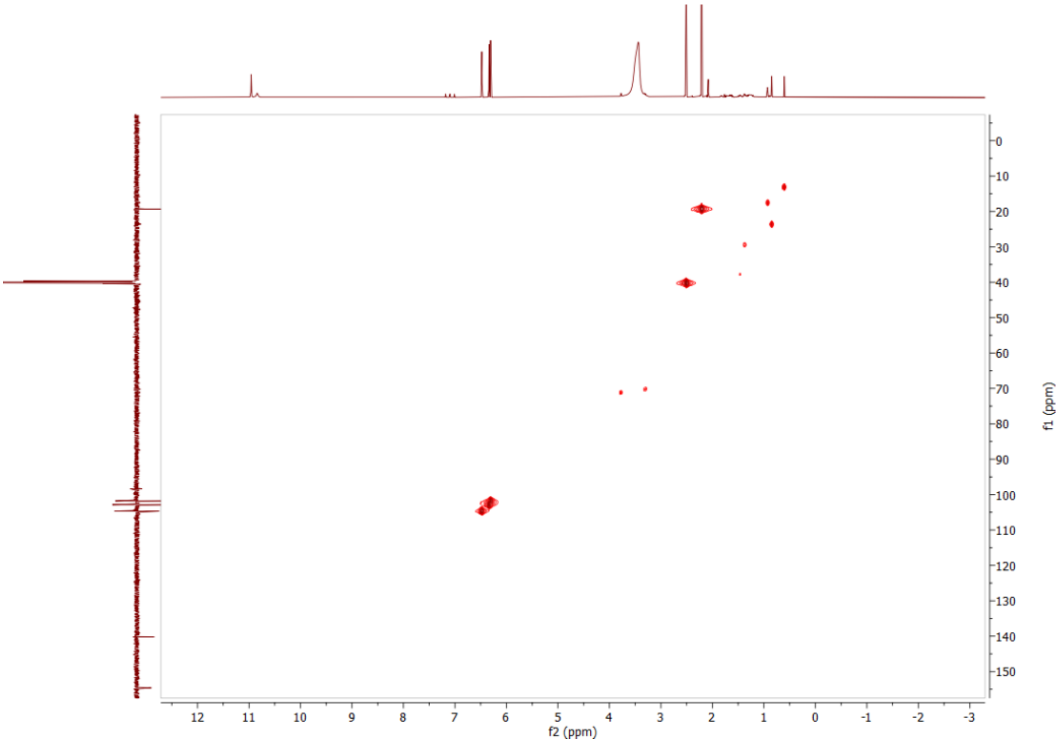

Product 4: 6-O-methylasparvenone

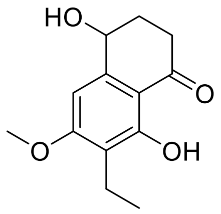

^{1}H NMR (600 MHz, DMSO)

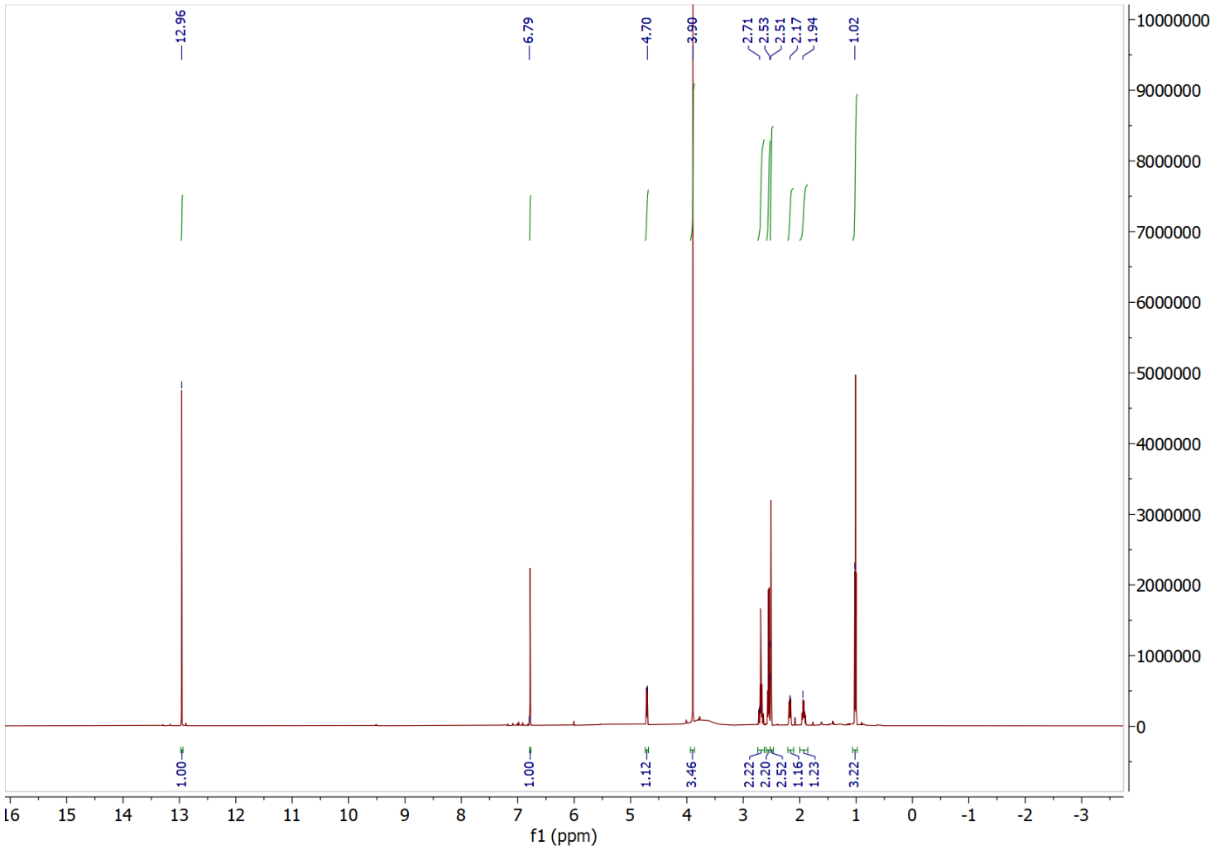

^{1}H NMR (600 MHz, DMSO) δ 12.96 (s, 1H), 6.78 (d, *J* = 0.9 Hz, 1H), 4.71 (ddd, *J* = 9.3, 4.1, 1.0 Hz, 1H), 3.90 (s, 3H, OC*H*$_3$), 2.76 – 2.62 (m, 2H), 2.55 (t, *J* = 7.4 Hz, 2H), 2.17 (dtd, *J* = 12.6, 5.2, 4.1 Hz, 1H), 1.93 (dtd, *J* = 12.6, 9.6, 5.4 Hz, 1H), 1.01 (t, *J* = 7.4 Hz, 3H, CH$_2$-C*H*$_3$).

^{13}C NMR (151 MHz, DMSO)

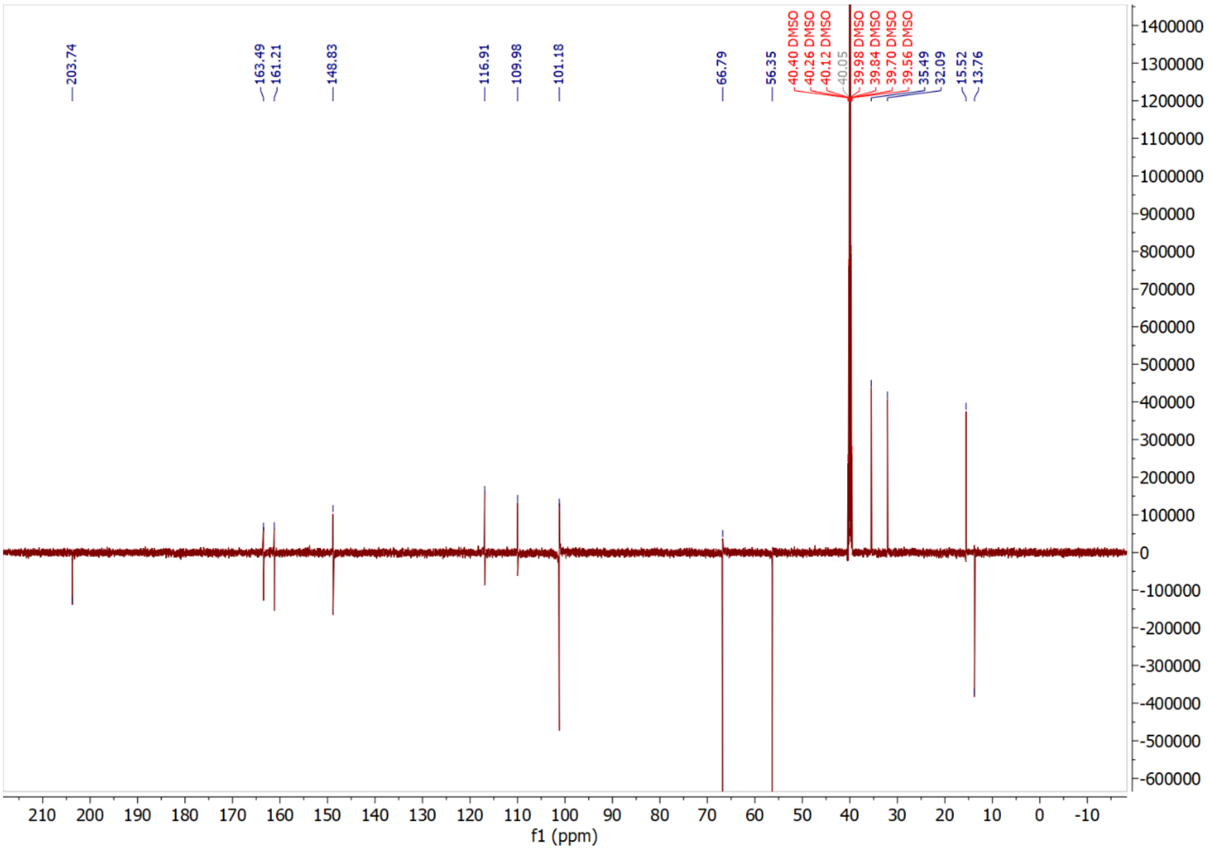

^{13}C NMR (151 MHz, DMSO) δ 203.74 (*C*O, carbonyl), 163.49 (*C*-OCH_3), 161.21 (*C*-OH, aromatic), 148.83, 116.91 (*C*H-CH_2-CH_3)., 109.98, 101.18 (*C*H, aromatic), 66.79 (*C*-OH, cyclohexane), 56.35 (O*C*H_3), 35.49 (*C*H_2, cycohexane), 32.09 (*C*H_2, cycohexane), 15.52 (*C*H_2-CH_3)., 13.76 (CH_2-*C*H_3).
