# Supplementary material for: Evolution-Informed Discovery of the Naphthalenone Biosynthetic Pathway in Fungi
Source: mBio. 2022 May 26;13(3):e00223-22. doi: 10.1128/mbio.00223-22 (PMC9239057; doi:10.1128/mbio.00223-22)
Supplement: FIG S3 [file mbio.00223-22-s0008.pdf]

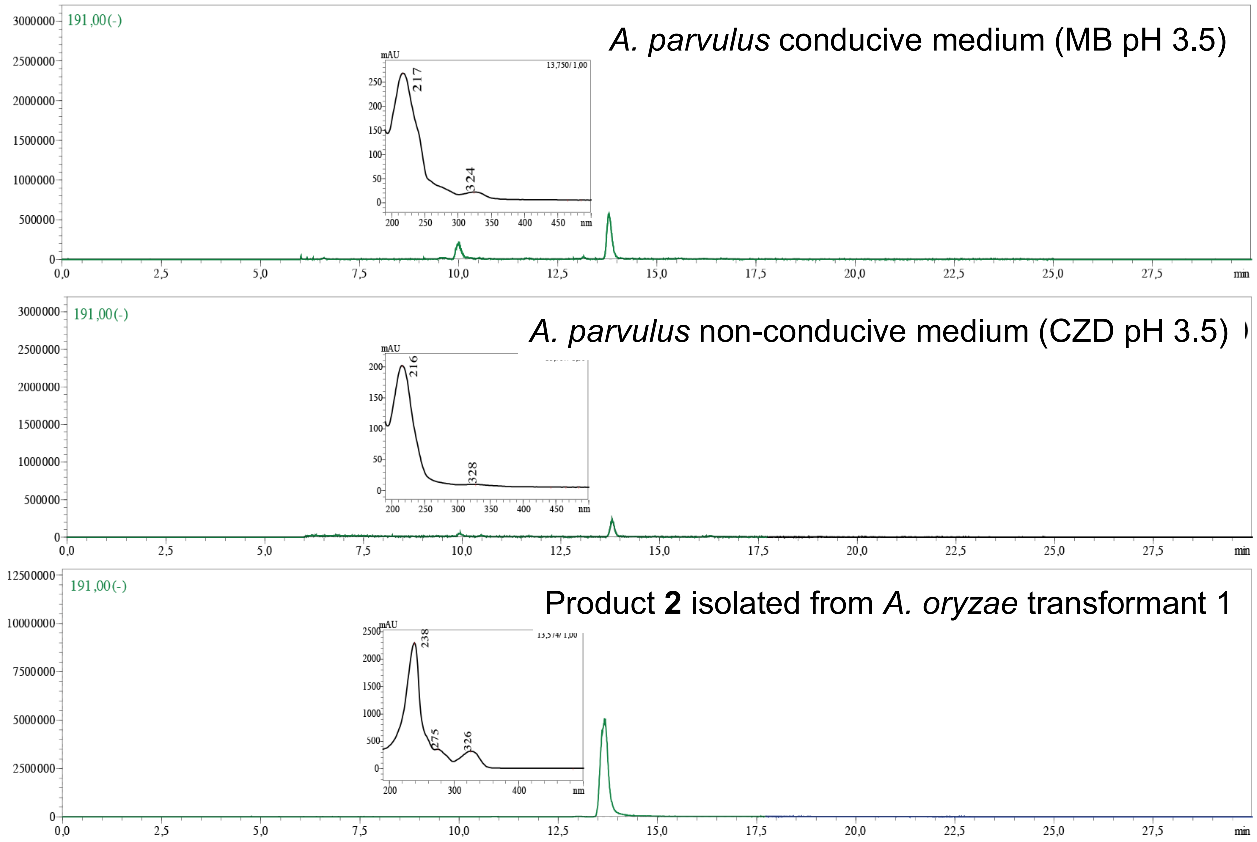

Fig. S3. Search for product 2 in organic extracts from *Aspergillus parvulus*. Extracted mass of product **2** (191 in negative mode) in extracts from conducive and non-conducive conditions, with UV spectra. Purified product **2** produced by *Aspergillus oryzae* transformant is showed as reference.
